# Haplotype-resolved chromosomal-level genome assembly reveals regulatory variations in mulberry fruit anthocyanin content

**DOI:** 10.1093/hr/uhae120

**Published:** 2024-04-23

**Authors:** Zhongqiang Xia, Wei Fan, Duanyang Liu, Yuane Chen, Jing Lv, Mengxia Xu, Meirong Zhang, Zuzhao Ren, Xuefei Chen, Xiujuan Wang, Liang Li, Panpan Zhu, Changying Liu, Zhiguang Song, Chuanshu Huang, Xiling Wang, Shuchang Wang, Aichun Zhao

**Affiliations:** State Key Laboratory of Resource Insects, Institute of Sericulture and Systems Biology, Southwest University, Chongqing 400715, China; State Key Laboratory of Resource Insects, Institute of Sericulture and Systems Biology, Southwest University, Chongqing 400715, China; State Key Laboratory of Resource Insects, Institute of Sericulture and Systems Biology, Southwest University, Chongqing 400715, China; State Key Laboratory of Resource Insects, Institute of Sericulture and Systems Biology, Southwest University, Chongqing 400715, China; State Key Laboratory of Resource Insects, Institute of Sericulture and Systems Biology, Southwest University, Chongqing 400715, China; State Key Laboratory of Resource Insects, Institute of Sericulture and Systems Biology, Southwest University, Chongqing 400715, China; State Key Laboratory of Resource Insects, Institute of Sericulture and Systems Biology, Southwest University, Chongqing 400715, China; State Key Laboratory of Resource Insects, Institute of Sericulture and Systems Biology, Southwest University, Chongqing 400715, China; State Key Laboratory of Resource Insects, Institute of Sericulture and Systems Biology, Southwest University, Chongqing 400715, China; State Key Laboratory of Resource Insects, Institute of Sericulture and Systems Biology, Southwest University, Chongqing 400715, China; State Key Laboratory of Resource Insects, Institute of Sericulture and Systems Biology, Southwest University, Chongqing 400715, China; Resource Institute for Chinese & Ethnic Materia Medica, Guizhou University of Traditional Chinese Medicine, Guiyang 550025, China; Key Laboratory of Coarse Cereal Processing, Ministry of Agriculture and Rural Affairs, Chengdu University, Chengdu 610106, China; Chongqing Sericulture Science and Technology Research Institute, Chongqing.400715, China; Chongqing Sericulture Science and Technology Research Institute, Chongqing.400715, China; College of Sericulture, Textile and Biomass Sciences, Southwest University, Chongqing, 400715, China; Institute of Environment and Plant Protection, Chinese Academy of Tropical Agricultural Sciences, Haikou 570100, China; State Key Laboratory of Resource Insects, Institute of Sericulture and Systems Biology, Southwest University, Chongqing 400715, China

## Abstract

Understanding the intricate regulatory mechanisms underlying the anthocyanin content (AC) in fruits and vegetables is crucial for advanced biotechnological customization. In this study, we generated high-quality haplotype-resolved genome assemblies for two mulberry cultivars: the high-AC ‘Zhongsang5801’ (ZS5801) and the low-AC ‘Zhenzhubai’ (ZZB). Additionally, we conducted a comprehensive analysis of genes associated with AC production. Through genome-wide association studies (GWAS) on 112 mulberry fruits, we identified *MaVHAG3*, which encodes a vacuolar-type H^+^-ATPase G3 subunit, as a key gene linked to purple pigmentation. To gain deeper insights into the genetic and molecular processes underlying high AC, we compared the genomes of ZS5801 and ZZB, along with fruit transcriptome data across five developmental stages, and quantified the accumulation of metabolic substances. Compared to ZZB, ZS5801 exhibited significantly more differentially expressed genes (DEGs) related to anthocyanin metabolism and higher levels of anthocyanins and flavonoids. Comparative analyses revealed expansions and contractions in the flavonol synthase (FLS) and dihydroflavonol 4-reductase (DFR) genes, resulting in altered carbon flow. Co-expression analysis demonstrated that ZS5801 displayed more significant alterations in genes involved in late-stage AC regulation compared to ZZB, particularly during the phase stage. In summary, our findings provide valuable insights into the regulation of mulberry fruit AC, offering genetic resources to enhance cultivars with higher AC traits.

## Introduction

The *Morus* genus, belonging to the Moraceae family, is found all over the world, but it is most common in the tropical and subtropical areas of the Northern Hemisphere. Because of their delicious flavor, eye-catching color, high nutritious content, and low-calorie content, mulberry fruits are widely grown throughout Asia. The cultivation area in China spans over 100 000 hectares, resulting in substantial seasonal harvests. Beyond its status as a consumable fruit, mulberry holds a historical legacy as both a dietary staple and a traditional medicinal herb [1]. Mulberries have various chemical constituents across their diverse types and maturation phases, including amino acids, fatty acids, minerals, polyphenols, and polysaccharides. Extracts obtained from mulberry fruits and their key bioactive elements, such as anthocyanins, rutin, and polysaccharides, have demonstrated a spectrum of biological activities, as evidenced by both *in vitro* and *in vivo* investigations [2]. Anthocyanins are the primary flavonoid compounds found in purple mulberries, with cyanidin-3-O-rutinoside (C3R) and cyanidin-3-O-glucoside (C3G) being predominant [3, 4]. Notably, compared to their yellow/white counterparts, red/purple fruits accumulate more anthocyanin [5]. The total AC significantly increases from the green/light red stage to the black background transition, accompanied by visible color changes [6, 7]. As a result, the color composition of mulberry fruit reveals valuable compounds and facilitates germplasm innovation.

Anthocyanins, renowned for their robust antioxidant attributes, have undergone a comprehensive exploration of their plausible human health benefits and anti-aging implications. The biosynthesis pathway and transcriptional regulatory intricacies governing anthocyanin pigmentation have been extensively studied in the context of crops, horticultural species, and model organisms [8–10]. Enzymatic reactions involving structural genes synthesize various modified forms of anthocyanins, such as glycosylation and acylation [11, 12]. These synthesized anthocyanins are transported to vacuoles for stable accumulation and storage in the cytoplasm [13, 14]. Regulatory factors affect the regulation of structural genes involved in anthocyanin biosynthesis [15], including the MBW complex [16, 17], ERF [18, 19], NAC [20], and WRKY [21, 22]. Furthermore, the pH of the vacuole affects the degree of color change [23], potentially influencing variations in AC among plant species and cultivars [24, 25]. Unlike fleshy fruits derived from ovary development, mulberries form through the thickening of the corolla and ovary walls of pistillate flowers, suggesting a different coloring mechanism [26]. Previous studies have identified genes and molecular mechanisms associated with mulberry fruit coloration, including pigment synthesis, transport, and degradation [27–29]. Notably, recent research has demonstrated that the MYBA-bHLH3-TTG1 transcription factor complex regulates anthocyanin biosynthesis with varying levels and proportions of anthocyanins, flavones, and flavonols found in mulberry fruits of different colors, such as purple, yellow, and white [30]. Despite notable advancements, substantial lacunae remain in comprehending the regulatory framework and pivotal genes dictating mulberry fruit color characteristics. This underscores the imperative of continued inquiry and extensive exploration in this domain.

Multi-omics integrative analysis has emerged as a powerful technique for the in-depth exploration of plant genomes, allowing breakthroughs in the mulberry industry by combining high-quality genomes with traditional breeding programs. Following the publication of the first mulberry genome of *Morus notabilis* [31], several high-quality chromosome-level genomes were made available [32–35]. However, the high heterozygosity, polyploidy, and extensive diversity of most mulberry varieties present challenges in unlocking the genome. Consequently, molecular-assisted breeding of elite cultivars and metabolic engineering of anthocyanins is hindered. Recent advances in PacBio HiFi read sequencing technology have enabled the generation of haplotype-resolved assemblies in tea plants [36], *Artemisia annua* [37], and kiwifruit [38]. These studies have greatly facilitated researchers in studying variety and species variations, obtaining more accurate chromosomal maps [39]. Integrative transcriptomic and metabolomic analyses are highly available for exploring new genes and critical biological pathways, providing a better understanding of the anthocyanin biosynthesis mechanism in mulberries. Whole-genome resequencing (WGS) of extensive germplasm collections has become feasible with the advent of low-cost and high-throughput next-generation sequencing technologies [40]. GWAS methods are commonly employed to identify genetic loci associated with traits, offering increased resolution. A combination of WGS and GWAS applied to complete genetic populations or diverse germplasm collections has been successfully employed in some crops [41–43].

We conducted sequencing of two diploid mulberries (*Morus*) cultivars, namely ZS5801 and ZZB. ZS5801, a valuable hybrid-derived variant, is notable for its prolific purple fruits. Conversely, ZZB, characterized by its ivory hue, exhibits exceptional sweetness and translucent white flesh resembling pearls, rendering it a valuable asset for varietal development. Our objective was to decipher the molecular underpinnings governing the accumulation of fruit color, a pivotal domestication trait in cultivated *Morus* trees, by employing a multi-faceted omics approach. Furthermore, we extended our scope by resequencing multiple color-representative mulberry fruits to scrutinize the genetic diversity of fruit color across distinct geographic populations and identify potential loci via association analysis. Our discoveries provide insightful perspectives on the mechanisms underpinning heterosis and fruit color buildup in mulberries, presenting a novel and comprehensive regulatory network.

## Results

### Sequencing, assembly, and annotation of haplotype-resolved genomes for ZS5801 and ZZB

**Figure 1:**
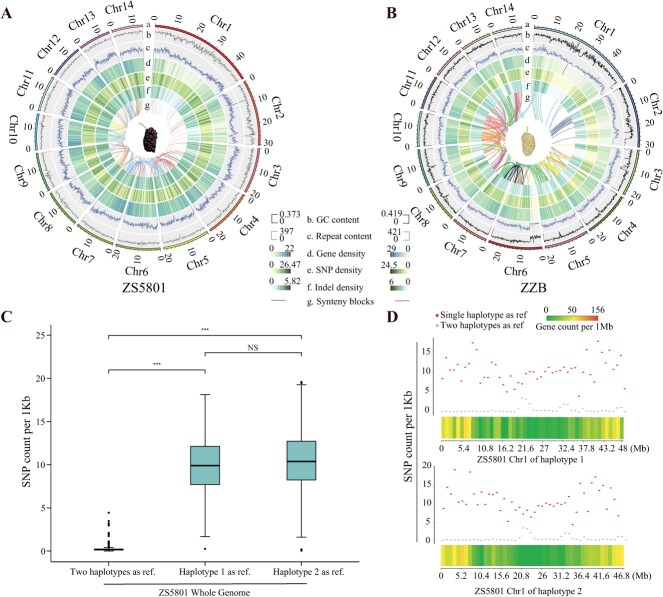
Genome assembly and annotation of ZS5801 and ZZB Haplotypes. **A** and **B** Circular diagrams illustrating the characteristics of haplotype 1 for ZS5801 and ZZB, respectively. From outer to inner tracks: (a) chromosome ideograms, (b) GC content (500 kb window size), (c) repetitive sequence content (500 kb window size), (d) gene density (500 kb window size), (e) SNP density (500 kb window size), (f) indel density (500 kb window size), and (g) interchromosomal synteny blocks. **C** From left to right: The first boxplot depicts SNP density in 1 Kb windows, using the fusion of two haplotypes as a reference. The second and third boxplots display the SNP density of haplotypes 1 and 2, respectively, with single haplotypes as references. When using a single haplotype as a reference, reads align to allelic regions, revealing heterozygous sites as SNPs and resulting in a notably higher SNP density than a single-haplotype reference. **D** Detailed representation of SNP density distribution for chromosome 1 in ZS5801 for the two haplotypes, employing distinct reference genomes.

We selected two representative mulberry cultivars in this study, ZS5801 and ZZB, showing notable AC variations for comprehensive genome sequencing. A high-depth sequencing approach was employed for ZS5801, incorporating 80$\times$ Illumina paired-end reads (PE150), 98$\times$ PacBio Hi-Fi reads, and 118$\times$ Hi-C data. Similarly, ZZB was subjected to sequencing involving 82$\times$ Illumina paired-end reads (PE150), 73$\times$ Hi-Fi reads, and 79$\times$ Hi-C data (Table S1, see online supplementary material). Initial insights into the overall genomic attributes were gleaned using high-depth Illumina paired-end reads (150 bp). Genome size estimation ranged between approximately 237 Mb and 256 Mb for both cultivars, accompanied by heterozygosity rates ranging from 1.27% to 1.45% (Fig. S1, Table S2, see online supplementary material). We conducted preliminary assembly by leveraging the hifiasm (v0.16.1-r375) [44] algorithm in ‘hic’ mode, yielding four haploid genomes for ZS5801 and ZZB (Fig. 1A and B, Table 1): ZS5801_Hap1, ZS5801_Hap2, ZZB_Hap1, and ZZB_Hap2, while retaining the primary assembly. The lengths of these haploid assemblies encompassed approximately 300 Mb (293.6–301.3 Mb) and 319 Mb (309–314.9 Mb), respectively. Notably, the contig N50 values ranged from 2.6 Mb to 8.9 Mb, indicating a high level of contiguity within the separated contig assemblies. Chromosome interaction maps aligned with the preliminary assembly were constructed using the HiC-Pro (v3.1.0) [45] program (Fig. S2, see online supplementary material). The interaction signals anchored each of the 14 pseudochromosomes to a distinct newly assembled contig, suggesting the absence of chromosome loss for all chromosomes.

**Table 1:** Statistics of the ZS5801 and ZZB genome assemblies

	**ZS5801**			**ZZB**		
	**Haplotype1**	**Haplotype2**	**Monoploid**	**Haplotype1**	**Haplotype2**	**Monoploid**
**Assembly**						
Genome size (bp)	301 384 141	293 602 840	302 793 785	309 049 771	314 939 203	319 777 161
Number of contigs	260	112	90	108	105	105
N50 of contigs (bp)	2 718 449	2 645 497	7 751 604	7 949 612	8 935 118	14 142 922
GC content of the genome	0.3476	0.3467	0.3478	0.3479	0.3485	0.3487
Complete BUSCOs	96.40%	97%	97%	96.80%	96.80%	97.30%
Chromosome-scale scaffolds (bp)	296 232 279	291 133 272	299 344 624	305 228 288	308 187 490	313 486 569
Anchor rate	98.29%	99.16%	98.86%	98.76%	97.86%	98.03%
LAI	24.13	23.78	19.78	11.99	18.4	11.31
Switch error rate (%)	6.42		7.59	7.93		8.33
**Annotation**						
Number of predicted genes	24 232	24 062	24 254	24 283	23 950	24 266
Average gene length (bp)	3527.94	3495.38	3511.84	3392.58	3442.9	3458.8
Average CDS length (bp)	1204.88	1207.03	1206.68	1206.78	1206.04	1213.52
Average exon number	5.02	5.04	5.04	5.01	5.05	5.94
Expression of the gene (TPM > 5)	19 000	18 969	19 011	19 408	19 068	19 160
Transcriptome support rate	0.78	0.79	0.78	0.80	0.80	0.79
Complete BUSCOs	96.40%	97.10%	96.70%	95.70%	96.60%	96.60%
Repeat sequences (bp)	158 551 299	151 528 403	159 954 253	167 260 334	172 107 648	177 161 311

The accuracy and reliability of the assemblies underwent thorough assessment through various methodologies. Initially, the precision of contig separation was verified by aligning short Illumina reads individually to single haplotypes and merged haplotypes of both genomes, followed by single nucleotide polymorphism (SNP) calling. Utilizing a solitary haplotype as a reference yielded a notably higher SNP density than employing both haplotypes (Fig. 1C and D; Fig. S3, see online supplementary material), attesting to the precise regional separation achieved within the assemblies. Moreover, our analysis unveiled an impressive 99.01% and 94.10% of Illumina paired-end short sequences displaying optimal pairing with the ZS5801 and ZZB assemblies, effectively accounting for 99.58% and 99.68% of assembly positions, respectively (Table S1, see online supplementary material). These results decisively confirm the comprehensive representation of sequence content within the assemblies.

Further evaluation of assembly integrity employed benchmarking universal single-copy orthologs (BUSCO) (v5.3.0) [46] analysis, which revealed a high level of completeness in genic regions, with 97% (1565/1614) in ZS5801 and 97.3% (1570/1614) in ZZB (Fig. S2, see online supplementary material). The assemblies successfully tackled complexities arising from intricate repetitive sequences. Assembly challenges often arise from highly complex repetitive sequences. The long terminal repeat (LTR) assembly index (LAI) [47] values for all four haplotypes exceeded 11.9 (Table 1), signifying their reference-grade quality and robust completeness, even in the presence of challenging repetitive elements. Telomeres were identified in the genomes of ZS5801 and ZZB using the conserved telomeric repeat sequence (5′-CCCTAAA/TTTAGGG-3′) as a query (Table S3, see online supplementary material). In ZS5801, eight telomeres were identified in haplotype 1, six telomeres in haplotype 2, and 11 telomeres in the monoploid genome. In ZZB, seven telomeres were identified in haplotype 1, eight telomeres in haplotype 2, and 10 telomeres in the monoploid genome. The approximate locations of the centromeres were also determined and recorded in Table S3 (see online supplementary material). To assess the switch errors between the two haplotypes of ZS5801 and ZZB, the calc_switchErr pipeline (https://github.com/tangerzhang/calc_switchErr/) was used. The switch error rate for ZS5801 was calculated to be 6.42% (25 083 out of 390 462), while the switch error rate for ZZB was 7.93% (39 420 out of 497 258) (Table 1). Previous studies have demonstrated that a high switch error rate (15%) can be observed in genomes with low sequence diversity (0.5%) [48]. In the case of ZS5801 and ZZB, their switch error rates were relatively low, which may be attributed to their higher heterozygosity rates (>1%). It is worth noting that the switch error rates can also be influenced by factors such as the contig assembly method and the process of Hi-C data scaffolding.

Repetitive sequences within the genomes spanned regions from 151 Mb to 159 Mb and 167 Mb to 172 Mb, constituting 51.61–52.61% of ZS5801 and 54.12–54.65% of ZZB (Table S4, see online supplementary material). Genome annotation employed an integrated approach, combining *de novo*, homology-based, and transcript-based predictions via the EvidenceModeler (v1.1.1) [49] (EVM) pipeline. This process yielded 23 950–24 283 high-confidence protein-coding gene models across the four haplotypes (Table 1). These models featured average gene and coding sequence (CDS) lengths of 5089.2 and 1416.3 base pairs (Table 1). Notably, approximately 96.45% of these predicted genes exhibited favorable BUSCO scores, with 18 969–19 408 genes (78.41–79.92%) supported by transcriptomic evidence. The latter displayed transcripts per million (TPM) values surpassing 5 per kilobase per million reads. This outcome underscores the robustness of gene predictions regarding completeness and accuracy. Furthermore, annotation coverage of approximately 95.4% was achieved for the predicted protein-coding genes using information from the non-redundant (NR, NCBI), SwissProt, and InterPro databases (Table S5, see online supplementary material). The annotation encompassed 475–834 tRNA genes, 443–1271 rRNA genes, and 936–969 other noncoding RNAs, including 126–130 miRNAs and 807–847 snRNAs (Table S6, see online supplementary material).

These results collectively establish the high integrity, continuity, and precision of the haplotype-resolved assemblies for the two mulberry varieties. These assemblies confidently facilitated subsequent downstream analyses.

### Genomic evolution and comparative analysis of ZS5801 and ZZB

**Figure 2:**
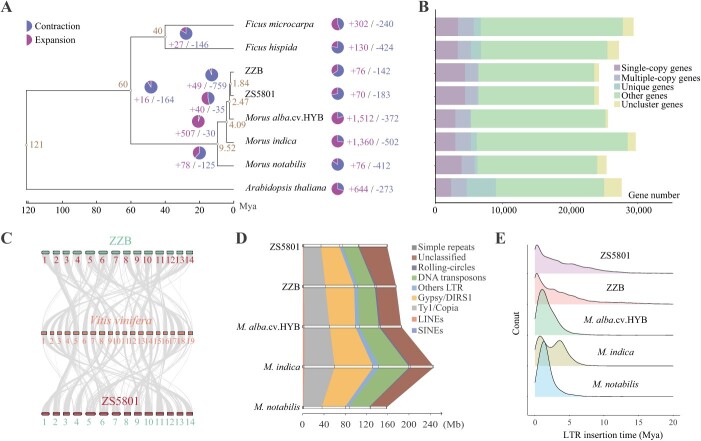
Evolutionary and comparative analysis of the Mulberry genome. **A** A phylogenetic tree was reconstructed using 3584 single-copy genes from mulberry species and three outgroup species. The OrthoFinder [50] identified orthologous gene families, and CAFE5 [51] calculated the count of expanded or contracted gene families, denoted by symbols (+ for expanded, − for contracted). Nodes on the phylogenetic tree include transparent circular plots indicating divergence times. **B** Clustering of orthologous and copy number gene families. Genes not clustered are from orthologous families with one gene each. Singlecopy genes are from families with one gene across eight genomes, while multi-copy genes are found in families with at least two genes in eight genomes. Unique genes are from families with genes exclusive to a single genome. **C** Genome collinearity analysis among ZS5801, grape, and ZZB. **D** Statistics on the size of repetitive sequences for each genome. **E** Comparison of long terminal repeat (LTR) insertion times across five mulberry genomes.

ZZB, a colorless mulberry variety native to northern China, is esteemed for its sweet flavor, while ZS5801, a pigmented mulberry cultivar, boasts enhanced yield and nutrient composition. Decoding the genetic makeup of ZS5801 holds promise for elevating AC, fruit production, and expanding cultivation areas. We compiled protein-coding genes from various sources, including *Arabidopsis thaliana*, banyan, and published mulberry genomes, to assess the phylogenetic relationship between ZS5801 and ZZB (Table S7, see online supplementary material). We elucidated their evolutionary dynamics by analysing 3584 single-copy gene families. The constructed maximum likelihood phylogenetic tree indicated a relatively recent divergence between ZS5801 and ZZB approximately 1.84 million years ago (Fig. 2A and B). Seventy gene families expanded, and 183 contracted, linked to processes such as protein ubiquitination, ATP-dependent enzymes, and defense responses during the speciation of ZS5801 (Fig. S4A and Table S7, see online supplementary material). In the ancestral development of ZZB, 76 gene families expanded, while 142 contracted, potentially connected to pollen recognition and salt stress responses (Fig. S4B and Table S7, see online supplementary material), reflecting their distinctive adaptability traits.

We identified 24 254, 26 246, and 24 266 pairs of orthologous genes between ZS5801, grape, and ZZB, respectively, using JCVI (https://github.com/tanghaibao/jcvi). Parameter distribution analysis suggested the absence of whole-genome duplication events in both genomes (Fig. 2C). Like many plants, mulberry genomes contain abundant repetitive sequences, predominantly transposable elements (TEs), with Copia and Gypsy LTR elements prevalent. Notably, ZZB possessed more intact LTR elements (80 077 in ZS5801 and 136 057 in ZZB) (Fig. 2D; Table S4, see online supplementary material). Both genomes exhibited recent insertion events (Fig. 2E) consistent with their recent divergence. Furthermore, ZZB displayed a denser pattern of LTR insertions, which was potentially linked to the higher count of intact LTR elements. This high-quality assembly provides insights into these evolutionary events.

### Assessing genetic diversity in the mulberry fruit population

Population genetic analysis provides a comprehensive understanding of the genetic factors associated with various phenotypes. Here we conducted an extensive investigation of genetic variations across the mulberry fruit population. A total of 112 individuals from diverse geographic regions in China, ranging from the south to the north, were included in the analysis (Table S8, see online supplementary material). Whole-genome sequencing (WGS) was performed on these individuals to capture a wide range of genetic information. The ZS5801_haplotype1 genome was used as the reference for variant calling. As a result, we identified a total of 3 205 768 SNPs with a minor allele frequency (MAF) greater than 0.05. The average sequencing depth achieved for the samples was 25.58$\times$ (Table S8, see online supplementary material).

**Figure 3:**
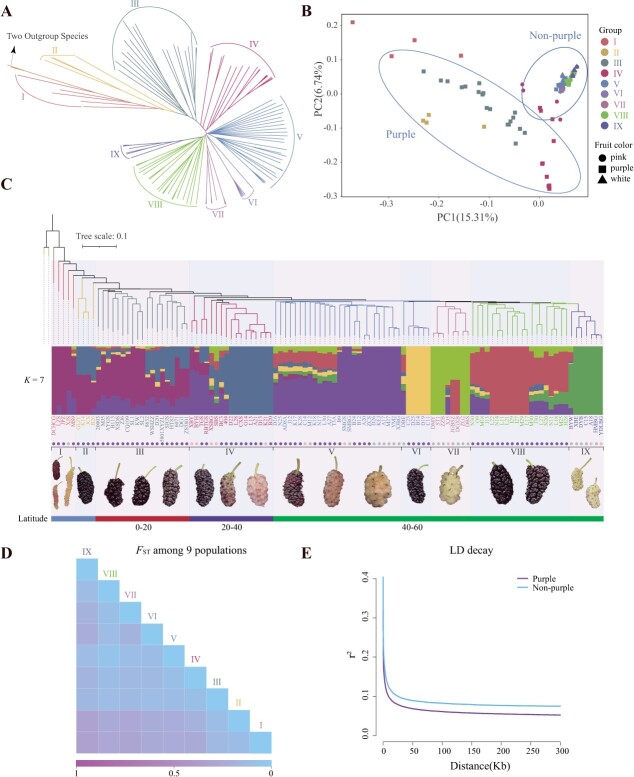
Population structure of mulberry fruit varieties. **A** Phylogenetic tree illustrating the relationships among 112 fruit mulberry accessions. **B** The principal component analysis (PCA) of the 112 fruit mulberry accessions revealed the population structure. Various subspecies are differentiated by unique symbols (circles, squares, triangles) which correspond to fruit color categories identified in (**A**). A line distinguishes the groups with purple fruit from those without. **C** ADMIXTURE analysis of nine fruit mulberry taxa, with representative mulberry types displayed beneath each group. **D** Genetic differentiation (*F*_st_) between each pair of subspecies, as indicated in (**A**). **E** Linkage disequilibrium (LD) attenuation for groups differentiated by fruit color characteristics discussed in (**B**).

These SNPs facilitated phylogenetic and principal component analyses (PCA) to discern the relationships among the 112 samples (Fig. 3A and B). The mulberry varieties delineated distinct branches (Fig. 3C), forming nine populations: (I) wild/near-wild populations, (II) primarily from Japan, (III) Guangdong province varieties, (IV) varieties concentrated in the 20–40 latitude band, and (V) to (IX) varieties mainly distributed within the 40–60 latitude band. These encompassed major cultivated mulberry species, including both purple and white varieties. Notably, the (VI) group originated from northeastern China, while the (VIII) and (IX) groups originated from northwestern and northern China, respectively. These groupings encapsulate the principal mulberry domestication and cultivation zones. Despite distinct phenotypic traits, including a pink phenotype in group (IV) and a white phenotype in groups (VII) and (IX), genetic clustering did not consistently align with color differentiation. ADMIXTURE [52] analysis corroborated these observations, identifying an optimal genetic cluster number (k) of 7 (Table S9, see online supplementary material).

**Figure 4:**
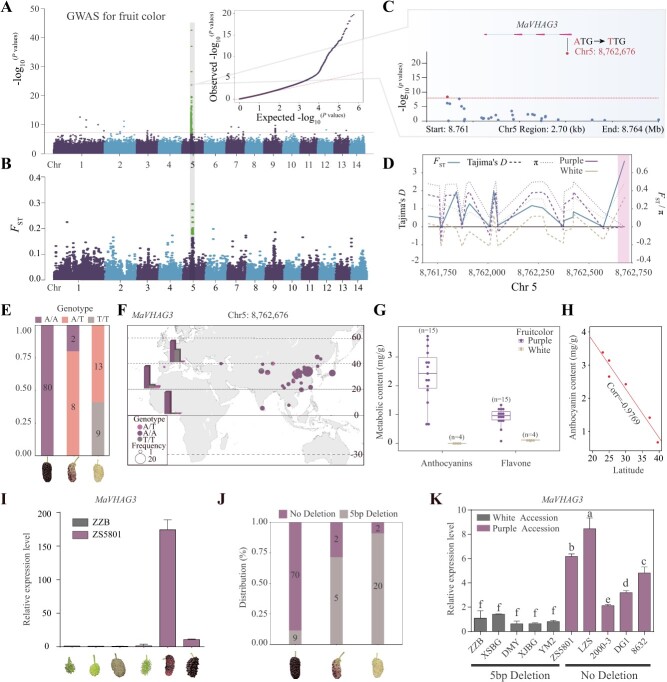
Identification of key loci for fruit color traits via GWAS. **A** Manhattan plot illustrating the results of GWAS for fruit color in 112 accessions, with data analyzed using a linear mixed model (LMM). A dashed line marks the significance threshold, set by Bonferroni correction (*P* = 1.11 × 10^−8^). The quantile-quantile plot displays -log_10_ transformed observed *P*-values against expected *P*-values. **B** Genome-wide *F*_st_ analysis depicted through a Manhattan map, showing genetic differentiation across the genome. **C** Gene structure of *MaVHAG3* within the haploblock containing the lead SNP. Exons are represented by boxes, introns by connecting lines, and gene orientation by arrows. **D** Comparative analysis of Tamjim’s *D*, *F*_st_, and π values for the *MaVHAG3* sketch region (−1 kb). Areas indicating potential selection signals are highlighted. **E** Allelic distribution in *MaVHAG3* genes among cultivars producing fruit with differing color traits, visually depicted alongside corresponding fruit images. Scale bars: 1 cm. **F** Geographical distribution of 112 accessions based on *MaVHAG3* genotypes, with the percentage ratio between different latitudes shown in the histogram. **G** Anthocyanin and flavonol content in the fruits of 19 accessions presented through box plots. **H** Correlation investigation between anthocyanin content (AC) and latitude in six mulberry cultivars, indicated by correlation coefficients. **I** qRT-PCR analysis of *MaVHAG3* expression during fruit development, quantifying dynamic changes in expression across diverse cultivars. **J** Percentage distribution statistics of promoter variation types in the three groups of cultivars. **K** Relative expression of different promoter variant types in 10 germplasms during mulberry fruit development, indicated by statistical significance using a one-way ANOVA test. Each accession underwent three replicates for robustness.

Pairwise *F*_st_ values quantified genetic divergence across the nine subgroups, with the highest values observed between Pop1 and Pop9, suggesting limited gene flow between these groups (Fig. 3D). Notably, genetic differentiation persisted even among closely related varieties, such as purple Pop6 and white Pop9, highlighting the influence of color-based selection. Linkage disequilibrium (LD) decay values were also computed for the samples, revealing faster LD decay in the purple variety compared to the white variety (Fig. 3E). These findings support the chronological divergence of the purple variety, which serves as the precursor for the white variety. Nucleotide diversity in the purple variety exceeded that in the white variety, potentially contributing to accelerated LD decay in the former.

### Selection of *MaVHAG3* genes governs mulberry color evolution

Fruit color, a vital agricultural trait in crops and fruit trees, remains incompletely understood in mulberry fruits. We conducted a GWAS using a linear mixed model (LMM) implemented in GEMMA [53], employing genotype and phenotype datasets from 112 mulberry individuals to elucidate the molecular underpinnings of mulberry fruit color evolution. We aimed to pinpoint the genomic loci responsible for fruit color modulation during domestication and breeding. A stringent significance threshold of 1.11 × 10^−8^ (Bonferroni correction) was set for *P*-values, revealing a prominent association signal on chromosome 5, encompassing 180 trait-associated markers (MTAs) (Fig. 4A). In search of candidate genes linked to mulberry fruit color, we scrutinized genes within a 10 kb window of the most significant SNP, covering at least 10% of the gene length and identifying 41 candidates. Integration with transcriptomic data further highlighted *ZS5801_Hap1.007690.1* as a key candidate (Fig. S5, Table S10, see online supplementary material). Phylogenetic analysis positioned *ZS5801_Hap1.007690.1* within the VHA subunit G gene family, with the closest relation to *A. thaliana*’s *VHAG3* protein, thus termed *MaVHAG3*. Notably, *MaVHAG3* demonstrated significant upregulation in ZS5801 during fruit ripening. Remarkably, a significant SNP in the last exon of *MaVHAG3* contained a Met110Leu missense mutation, highly linked to fruit color variation (Chr5: 8762676, A > T, *P*-value = 1.72 × 10^−24^) (Fig. 4C). These findings identify *MaVHAG3* as a credible candidate for improving mulberry fruit color, a pivotal determinant of fruit flavor quality.

Subsequently, we assessed the nucleotide sequence diversity of *MaVHAG3*, *F*_st_, and Tajima’s *D* (Fig. 4B and D). Notably, the purple population exhibited heightened diversity values at the mutation sites, and both color populations displayed pronounced signs of selection. We comprehensively examined this variation across mulberry germplasms to discern whether this variation arose during domestication. Notably, *MaVHAG3* harbored a significant single SNP (A > T) in the mulberry population. Varieties with the A/A genotype were mainly found in groups (I), (II), and (III), while the T/T genotype predominated in groups (VII) and (IX) (Fig. 4E; Table S11, see online supplementary material). Intriguingly, T/T genotype materials exhibited lower AC and higher latitude values (Fig. 4F). The AC and latitude displayed a negative correlation (Pearson correlation coefficient = −0.9769) in a selected subset of six varieties (Fig. 4G and H). The allele frequency of the single SNP and nucleotide sequence diversity within *MaVHAG3* suggest that the natural variation of *MaVHAG3* alleles might have been subject to selection during mulberry population domestication, potentially correlated with latitude.

We uncovered the significant influence of a single SNP variation within *MaVHAG3* on anthocyanin accumulation in mulberry fruits through RNA-seq and qRT-PCR analyses (Fig. 4I; Fig. S5B, see online supplementary material). *MaVHAG3* expression levels exhibited pronounced disparities between the colorless and purple varieties, indicating mutation-induced changes in the regulatory region of the gene. Subsequent examination of *MaVHAG3*’s upstream 2 kb promoter sequence unveiled a 5-bp Indel (90.90%) in the promoter region of colorless mulberry fruits (Chr5: 8760137), whereas the majority of purple mulberry fruits lacked this deletion variation (89.74%) (Fig. 4J; Table S12, see online supplementary material). qRT-PCR results further confirmed that the expression level of the *MaVHAG3* gene with the deletion variation in the promoter was significantly lower in white mulberry varieties compared to nonvariant purple fruit varieties (Fig. 4K). These findings underscore the functional impact of the 5-bp indel on the promoter, leading to divergent expression patterns of *MaVHAG3*.

Our study highlights *MaVHAG3* as a pivotal candidate gene that orchestrates anthocyanin accumulation in mulberry fruit. The naturally occurring genetic variations encompassing a single SNP within the coding region and a 5-bp indel within the promoter region are essential causal loci underpinning this trait.

### Dynamic regulation of flux synthesis enzymes governing fruit AC.

**Figure 5:**
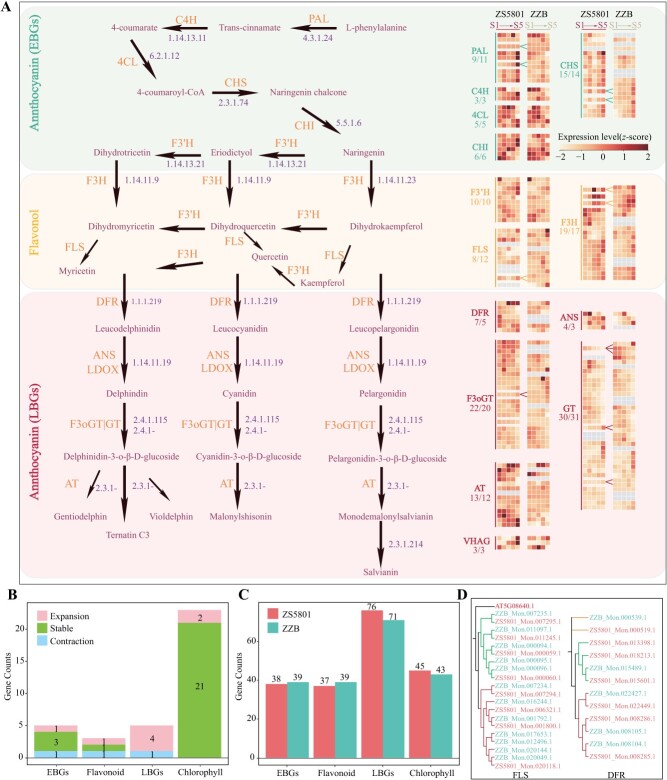
Metabolic pathways and time-ordered gene expression of anthocyanin and flavonol biosynthesis in ZS5801 and ZZB. **A** Heat map illustrating gene expression values from RNA-seq data across fruit developmental stages (labeled S1–S5) for both cultivars. Genes are categorized into three groups: ‘flavonol biosynthesis’, ‘early anthocyanin biosynthesis’, and ‘late anthocyanin biosynthesis’. Key enzymes highlighted include 4CL (4-Coumaric acid CoA ligase), ANS (anthocyanin synthase), AT (plant anthocyanidin transferase), C4H (cinnamate-4-hydroxylase), CHI (chalcone isomerase), CHS (chalcone synthase), DFR (dihydroflavonol reductase), F3H (flavanone 3-hydroxylase), F3’H (flavonoid 3′-hydroxylase), F3oGT (flavonol-3-oglucosyltransferase), FLS (flavonol synthase), GT (glycosyltransferases), PAL (phenylalanine ammonialyase). **B** Histogram displaying counts of enzyme families across anthocyanin, flavonol, and chlorophyll metabolic pathways, with categories labeled as ‘expanding’, ‘stable’, or ‘contractile’ based on gene fraction criteria. **C** Count of genes within different metabolic pathways for anthocyanins, flavonols, and chlorophyll. **D** Phylogenetic tree depicting FLS and DFR gene families within the ZS5801 and ZZB genomes, with gene IDs distinctively marked for each cultivar.

To deepen our understanding of the regulation of elevated AC, the metabolic pathways driving the formation of fruit colors have been reconstructed. We successfully identified the enzyme genes involved in this process (Fig. 5A; Fig. S6, Table S13, see online supplementary material). We predicted anthocyanin and flavonol biosynthetic enzymes by comparing the anthocyanin synthesis pathways between ZS5801 and ZZB. Flavonol synthesis forms anthocyanins and colorless flavonols through a branching pathway (Table S14, see online supplementary material). Notably, we identified a genome-wide absence of the F3’5’H gene.

The comparative analysis demonstrated differential expansion and contraction (six expansions and three contractions) within nine out of 13 anthocyanin biosynthesis pathway families between the two cultivars (Fig. 5B). This contrasted with the relatively few families (8%) within the chlorophyll metabolism pathway. Flavonol shares seven biosynthetic steps with anthocyanins, known as early anthocyanin synthesis (EBGs), and exhibits active homologous gene expression. However, the cultivars exhibited substantial differences in the number of pathway enzyme genes (76 and 71, respectively) during late-stage anthocyanin synthesis (LBGs), leading to a significant divergence in the overall expression patterns (Fig. 5C).

Crucially, two enzymes, DFR and FLS, showed notable expansion and contraction (7:5 and 8:12, respectively) (Fig. 5D). The contracted DFR gene’s total expression decreased significantly, while the expression of the expanded FLS gene increased notably (Fig. S7, see online supplementary material). These enzymes act as rate-limiting factors during anthocyanin and flavonoid synthesis. Changes in enzyme numbers could alter carbon flux within the flavonol pathway, consequently affecting color accumulation.

### Regulation of temporal gene expression during fruit development

**Figure 6:**
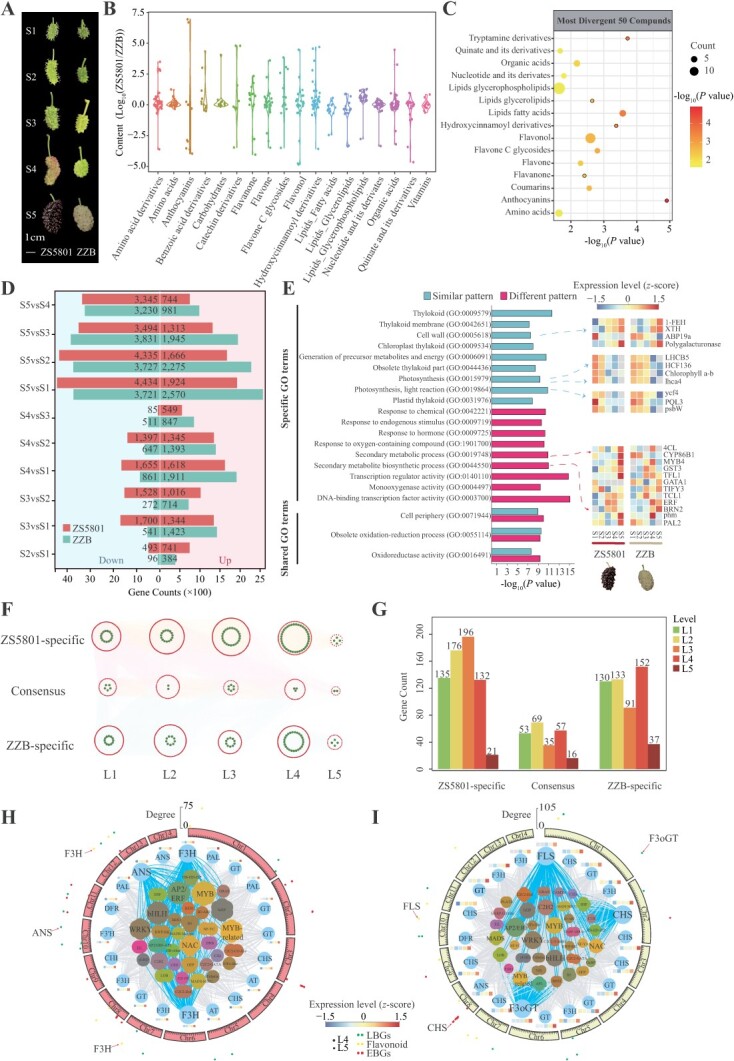
Transcriptional and metabolic patterns associated with fruit color. **A** Developmental phenotypes (stages S1–S5) of the ZS5801 and ZZB fruits. **B** Metabolite content analysis in ripe mulberry fruit of ZS5801 and ZZB. **C** Differential metabolic enrichment in ripe mulberry fruits of ZS5801 and ZZB. **D** Differential expression genes (DEGs) analysis between the five developmental time points of ZS5801 and ZZB. **E** Gene ontology (GO) enrichment analysis results for homologous genes with distinct and similar DEG expression patterns. Patterns with differing and matching expression are distinctly marked. **F** Predicted gene co-expression networks illustrating connections between transcription factors and enzyme genes in the anthocyanin/flavonol biosynthetic pathway. Networks are denoted by different levels (L1 to L5), indicating ZS5801-specific, ZZB-specific, and consensus temporal gene co-expression networks (TOGCNs). Connections between enzyme genes are not shown. **G** Quantification of co-expression network gene sets aligned with network diagrams in (**F**). **H** and **I** Specific subnetworks during the S4 and S5 stages in ZS5801 and ZZB: Heatmap analysis of average TPMs (z-score normalized) for core genes at distinct fruit development time points. Metabolic pathways are distinguished by varying patterns and symbols. Node degree in the TO-GCN indicates interaction frequency, with higher values denoting core status. Specific and core enzyme/transcription factor interactions are differentiated by line styles.

Considering the temporal-scale linear or nonlinear dynamics inherent in AC, we have implemented a multi-omics investigation that spans across multiple developmental stages. Here, we combined transcriptome and metabolome to conduct an in-depth analysis of the mulberry fruit color regulation. Distinct color variations were observed in the ZS5801 and ZZB throughout fruit development (Fig. 6A). ZS5801 accumulated anthocyanins, leading to a purple hue, while ZZB exhibited gradual chlorophyll degradation and turned jade white. Metabolite profiling indicated significant differences between high anthocyanin-yielding (HG2) and low anthocyanin-yielding (BYW) lines [27], particularly in flavonol content. Untargeted metabolic analysis of ripe fruits using liquid chromatography-mass spectrometry (LC–MS/MS) revealed the identification of 543 metabolites, including carbohydrates, organic acids, and amino acids, contributing to fruity flavors (Fig. 6B; Table S15, see online supplementary material). The detected metabolites associated with anthocyanin accumulation, such as cyanidin 3-O-glucoside, quercetin 3-O-rutinoside, and others, exhibited significant differential accumulation between ZS5801 and ZZB (Fig. 6C), consistent with previous findings [27]. Furthermore, we aimed to gain new insights into gene expression and transcriptional regulation during fruit development by analysing 30 RNA-sequencing datasets from five fruit developmental stages of ZS5801 and ZZB. A total of 18 025 genes were found to exhibit differential expression (|log_2_[fold change] | ≥ 2 and corrected *P*-value <0.05) across the five tissues and two cultivars. Among these genes, 12 260 and 14 443 were upregulated, while 22 466 and 17 437 were downregulated (Fig. 6D). We classified the expression patterns of differentially expressed genes (DEGs) into eight clusters according to their expression patterns, demonstrating either similar or opposite expression trends between the two cultivars (Fig. S8, see online supplementary material).

Specifically, 2049 homologous genes exhibited similar expression patterns, whereas 3822 genes displayed divergent expression patterns. Enrichment analysis revealed that both cultivars showed consistent differential expression trends in genes related to cell wall metabolism (GO:0005618, *P*-value = 3.09E-08) and photosynthesis (GO:0019864, *P*-value = 4.50E-10), while showing opposite trends in genes associated with anthocyanin, flavonol, and other secondary metabolic pathways (GO:0019748, *P*-value = 1.09E-11) (Fig. 6E). Moreover, utilizing an additional set of 32 transcriptome datasets, we constructed a gene co-expression network using the weighted gene co-expression network analysis (WGCNA) software package [54]. This resulted in the formation of 25 modules comprising 22 807 genes. Notably, the orange-red module, which included the anthocyanin synthase gene as a central hub, contained 110 additional genes (Table S16, see online supplementary material). Functional enrichment analysis of these genes revealed significant associations with secondary metabolism and flavonoid synthesis, indicating their potential involvement in fruit color formation (Fig. S9, see online supplementary material).

Using identified anthocyanin pathway enzymes and transcription factors, we constructed time-ordered gene co-expression networks (TO-GCNs) for two mulberry cultivars (Table S17, see online supplementary material). These networks were compared to unravel the gene regulatory mechanisms governing the fruit color transition between the cultivars. Among the 1624 genes exhibiting significant expression differences across the five developmental time stages (S1–S5) and with an average TPM value greater than 0.5, a basic helix–loop–helix (bHLH) transcription factor (*ZS5801_Monoploid.003909.1*) with a characteristic expression pattern was selected as the initial node. ZS5801-specific, ZZB-specific, and Consensus TO-GCNs were reconstructed (Fig. 6F), each comprising five temporal subnetworks (L1–L5) corresponding to the sampling time points. Genes associated with anthocyanin/flavonol biosynthesis pathways were identified within these networks: 657 genes (562 transcription factors and 95 pathway enzymes) in ZS5801-specific, 540 genes (480 transcription factors and 60 pathway enzymes) in ZZB-specific, and 230 genes (212 transcription factors and 18 pathway enzymes) in Consensus TO-GCNs (Fig. 6G).

Species-specific networks revealed crucial regulatory genes and their hierarchical interactions. In the late coloring stages (S4–S5), the ZS5801-specific subnetwork predicted critical anthocyanin pathway genes (F3H and ANS), while the ZZB-specific subnetwork highlighted CHS, FLS, and F3oGT genes (Fig. 6H and I; Fig. S10, see online supplementary material). These genes were validated through expression profiles correlated with color accumulation patterns during the critical transition and ripening stages. Interaction patterns between pathway enzymes and regulatory transcription factors were outlined, where the expression profiles of transcription factors (e.g., MYB, bHLH, WD40, ERF, and WRKY) closely aligned with color accumulation patterns, affirming the network’s accuracy. These network topologies lay the groundwork for further exploration of anthocyanin regulation.

## Discussion

A key agronomic trait sought by breeders in mulberry plants is high AC. Mulberry fruits exhibit significant variations in AC across different genotypes [55, 56], resulting in diverse mature fruit colors such as black, pink, red, and white [57]. This natural diversity among mulberry genotypes is essential for studying the metabolic pathways involved in anthocyanin biosynthesis and understanding the fundamental compounds that influence variations in AC among different mulberry types. These insights have the potential to streamline the screening of various germplasms and inform agricultural practices focused on anthocyanin-related applications.

Through the identification of these differences, specific loci and candidate genes associated with fruit color determination have been pinpointed. One of the identified candidate genes is *MaVHAG3*, which encodes a VHA transporter protein. This protein plays a crucial role in cellular functionality and coloration in fleshy fruits and ornamental plants. Processes such as anthocyanin and malic acid transportation rely on the activity of VHAs and vacuolar H^+^-pyrophosphatases, which are responsible for H^+^ pumping. In apples, the *MdMYB1* gene has been found to activate VHA B-subunit genes (*MdVHA-B1* and *MdVHA-B2*) through promoter binding, enhancing VHA activity to regulate cellular pH and anthocyanin accumulation [58]. These findings highlight the applicability of our dataset and help precise insights that support genetic enhancements in coloration.

An intriguing discovery materialized in a 5 bp variation within the *MaVHAG3* promoter, closely associated with mulberry population AC. Additionally, this 5-bp sequence is absent in early domesticated Japanese and Guangdong mulberry varieties, while a 5-bp insertion characterizes several recently domestic white mulberry materials. These observations posit the potential role of the identified genetic variation in domestication. Moreover, a salient inverse correlation materialized between *MaVHAG3* variation and latitudinal position, thereby augmenting our comprehension of the geographic impetus shaping genetic heterogeneity. Our dissection of the *MaVHAG3* promoter sequence (Fig. S11, see online supplementary material) uncovered many light-responsive essential elements. This prompts speculation regarding the potential influence of varying light intensities at different latitudes on anthocyanin accumulation, conceivably mediated by light-responsive interactions with the *MaVHAG3* promoter region.

Furthermore, high AC in mulberry is the result of multi-level regulation. Varieties such as ZS5801 and ZZB exhibit significant differences in fruit color. By utilizing PacBio HiFi long reads and Hi-C data, we have successfully achieved haplotype phasing, providing valuable insights into the heterozygous genome landscape of mulberry. The dynamic expression of candidate gene is speculated to play a pivotal role in plant development and adaptation, but further investigation is needed to determine their precise molecular functions. Comprehensive investigations are warranted to elucidate their involvement in fruit anthocyanin accumulation.

## Materials and methods

### Plant materials

Genomic sequencing involved the analysis of young leaves from individual plants of two diploids (2n = 2x = 28) mulberry cultivars, ZS5801 and ZZB, characterized by distinct fruit coloration. To investigate transcriptomic and chemical dynamics related to fruit color development, we harvested fruit tissue samples at five developmental stages from three mulberry cultivars (ZS5801, ZZB, and CQ109) based on discernible external traits. To facilitate transcriptome sequencing, 45 tissue samples (comprising three replicates per time) were obtained across these five-time points. Furthermore, mature mulberry fruits (S5 stage) from ZS5801 and ZZB, encompassing three replicates each, were gathered for metabolome comparative analyses. To explore population-based fruit color variations, comprehensive WGS was conducted using 112 distinct materials from diverse geographic locations, predominantly in China. This set comprised 54 re-sequenced specimens and 58 samples procured from GenBank (Accession Number PRJNA597170) [59]. All sequencing subjects were cultivated and maintained within mulberry germplasm nurseries at Southwest University and Chongqing Sericulture Research Institute. Following collection, freshly harvested tissues were promptly cryopreserved in liquid nitrogen.

### Sequencing

#### Genome sequencing

Illumina Short-Read Sequencing: Genomic DNA was extracted using a CTAB-based protocol as previously described [60]. Sequencing on the Illumina HiSeq platform utilized a 350 bp insert size library, generating high-quality 150 bp paired-end reads. Following sequencing, raw data underwent trimming and filtering procedures.

PacBio Sequel II Sequencing: DNA samples were outsourced to AnnoRoad, a reputable genomics service provider. CCS libraries were created using the Pacific Biosciences method, producing two distinct Hi-Fi libraries with 20 kb insert sizes for the cultivars ZS5801 and ZZB. Sequencing was performed on the Pacific Biosciences Sequel II platform.

Hi-C Sequencing: Employing the Hi-C method, we crosslinked DNA and proteins to capture spatial DNA conformation. *MboI* restriction endonuclease was used for crosslinked young leaves, followed by enzymatic digestion, biotin labeling, ligation, and extraction to create a high-quality Hi-C library. The library underwent 12–14 PCR amplification cycles before sequencing on the Illumina HiSeqPE150 platform.

#### Transcriptome sequencing

RNA-seq libraries with 350 bp insert sizes were generated using the Illumina TruSeq RNA Library Preparation Kit. Sequencing was performed on the Illumina NovaSeq 6000 platform to reveal the complex transcriptional landscape. Total RNA extraction utilized the RNAprep Pure Plant Kit (Tiangen, Beijing, China), ensuring RNA integrity for subsequent library preparation.

#### Metabolome detection

Biological samples were vacuum freeze-dried using a Scientz-100F lyophilizer and then ground to powder form with a Retsch MM 400 grinder (30 Hz, 1.5 min). Subsequently, 50 mg of sample powder was combined with −20°C pre-cooled 70% methanolic aqueous internal standard extract (1200 μL extractant per 50 mg sample). Vortexing occurred every 30 min for 30 sec, repeated six times. After centrifugation (12 000 rpm, 3 min), the supernatant was filtered through a 0.22 μm microporous membrane and stored for UPLC–MS/MS analysis.

#### Genome size and heterozygosity estimation

Genome size and heterozygosity were assessed utilizing Illumina short-read data corresponding to specific cultivars. Filtered sequencing reads were subjected to k-mer frequency computation (K = 17–49) through Jellyfish (v1.1.10) [61] software. The Perl scripts available at https://github.com/josephryan/estimate_genome_size.pl were utilized to divide the total k-mer count by the peak value of the k-mer distribution, yielding the genome size estimate. GenomeScope, an online tool (http://qb.cshl.edu/genomescope/genomescope2.0/), was employed for presenting and enhancing the visualization of the genome size estimation results.

### Genome assembly and evaluation of ZS5801 and ZZB

#### Genome assembly strategy

Hifiasm (v0.16.1-r37546) [44], available at https://github.com/chhylp123/hifiasm, was utilized to perform genome assembly for ZS5801 and ZZB. The assembly process employed the following parameters: ‘—h1 read1.fq.gz —h2 read2.fq.gz HiFi-reads.fq.gz’. This resulted in the generation of a pair of assemblies that are resolved at the haplotype level, utilizing paired-end Hi-C reads. The initial assembly process yielded two distinct outputs: (i) the primary assembly (referred to as p_ctg in hifiasm) capturing an unpurged hybrid haploid, and (ii) the alternative assembly (referred to as a_ctg in hifiasm) encompassing missing alternative haplotypes from the primary assembly. For subsequent analyses, encompassing comparisons of metabolic pathway enzymes and genome-to-genome alignments, we generated a ‘haploid’ genome assembly outcome and assembly fragments representing heterozygosity. Eventually, we obtained a primary genome assembly and two draft haploid contig genomes.

#### Refinement and chromosome-level structure

Two iterations of Pilon (v1.24) [62] were employed for genome polishing, integrating resequencing data. The trimmed Hi-C raw data underwent analysis using Juicer (v1.6.2) [63], followed by the application of the juicerbox pipeline for manual misassembly correction. Subsequently, the genome was scaffolded into chromosome-level structures utilizing 3D-DNA [64] software, with parameters ‘-m haploid -I 15000 -r 0’.

#### Genomic assessment

The quality of genome assembly underwent comprehensive evaluation employing diverse datasets.

Verification of haplotype phase

Raw genomic DNA Illumina reads from the ZS5801 library (insert size: 250 bp) were aligned to reference genomes of Haplotype 1 (14 chromosomes), Haplotype 2 (14 chromosomes), and the two haploid chromosomes (totaling 28 chromosomes). BWA-MEM (v0.7.17-r1188) [65] with default settings facilitated the alignment. Subsequent SNP detection was carried out using BCFtools (v1.15) (http://samtools.github.io/bcftools/). SNP density (SNPs per 100 bp) was quantified through a 2 Mb sliding window analysis.

Mapping efficiency

Illumina reads were mapped to ZS5801 and ZZB assemblies using BWA-MEM (v0.7.17-r1188) [65] for resequencing data mapping. Qualimap2 (v2.2.1) [66] calculated mapping rates and coverage. The results indicated a successful alignment rate of 97.90% and 99.50% for the genomes, affirming robust assembly completeness. RNA-Seq data was filtered with Trimmomatic (v0.38) [67] and subsequently mapped to the assembled genomes using HISAT2 [68] with default parameters.

BUSCO completeness evaluation

Genome assembly completeness was assessed at both contig and chromosome levels using the Embryophyta odb10 database (version 2020-09-10), comprising 40 species and 1614 single-copy genes. The evaluation utilized BUSCO (v5.3.0) [46] software.

LTR analysis and LAI calculation

LTR structures were identified, and the LAI [47] was computed. This index serves as an indicator for gauging assembly quality for repetitive sequences.

Detection of centromere and telomere sequences

Telomere identification and centromere prediction were conducted using the quarTeT software (v1.1.6) [69]. The quartet_teloexplorer.py script with default parameters was used to directly search for the telomere sequence (5′-CCCTAAA/TTTAGGG-3′). Genome tandem repeats in the genome were identified using the quartet_centrominer.py script with default parameters. The centromeric tandem repeat was determined as the continuous and most abundant repeat unit [70].

Estimation of switch errors between haplotypes

To calculate the switch error rate, we refer to the method proposed by Zhang *et al.* [36]. The calc_switchErr pipeline, available at the GitHub repository (https://github.com/tangerzhang/calc_switchErr), was utilized to calculate switch errors between two haplotypes, ZS5801 and ZZB.

### Repeat elements and gene prediction

#### Prediction of repeating sequences

Genomic repeat prediction employed RepeatMasker (v4.1.2-pl) and RepeatProteinMask (v4.1.2-p1) [71] software along with default parameters of Tandem Repeats Finder [72], LTR Finder [73], and RepeatScout [74] for *de-novo* prediction. LTR-RT annotation utilized LTRharvest [75] from GenomeTools (v1.5.9). LTRharvest outputs were analysed via LTR_retriever (v2.8) [76] to ascertain LAI and LTR-RT insertion ages.

#### Gene prediction

Three approaches were adopted for protein-coding gene structure prediction: *de novo*, homology-based, and RNA-seq-based prediction. Before gene prediction, the assembled genome underwent hard- and soft-masking using RepeatMasker (v4.1.2-p1) [71]. (i*) Ab initio* prediction involved Augustus v3.4.0 [77] and GlimmerHMM [78] for coding region prediction. (ii) Homology-based prediction utilized GeMoMa [79], mapping cds sequences from *A. thaliana*, *Cannabis sativa*, *Ficus microcarpa*, *F. hispida*, *Fragaria vesca*, *Malus domestica*, *Morus notabilis*, *Nicotiana tabacum* L., *Oryza sativa*, *Prunus persica*, ZZB and ZS5801 to the genome assembly. (iii) RNA-seq-based prediction employed Trinity (v2.13.2) [80] for transcriptome assembly, followed by PASA [81] for gene prediction. TransDecoder (v5.5.0) (https://github.com/TransDecoder) software to identify potential coding regions in the transcription sequence. EVidenceModeler (v1.1.1) [49] integrated predictions from the three methods to yield a comprehensive non-redundant gene set. Weighted points were assigned based on the reliability of the software: GeMoMa 5 points, Augustus 3 points, GlimmerHMM 1 point, SNAP 1 point, PASA 7 points, and TransDecoder 10 points. For protein annotation, InterProScan (v5.35–74.0) [82] and BLASTP [83] searches against KEGG, Swiss-Prot [84], and TrEMBL databases were performed with an e-value threshold of 1e^−5^.

#### Noncoding RNA identification

Noncoding RNAs were detected using Rfam (v14) [85] and Infernal (v1.1.2) [86]. tRNAs were recognized with tRNAscan-SE (v2.0.8) [87] using default parameters. rRNA sequences were predicted through RNAmmer [88], while snRNAs were identified via Infernal [86] from the Rfam database.

#### Comparative genomic analysis

Non-redundant protein sequences from eight species, namely *A. thaliana*, *F. microcarpa*, *F. hispida*, *M. notabilis*, M.alba.cv.HYB, *Morus indica*, ZS5801, and ZZB, underwent phylogenetic analysis. *A. thaliana* data was retrieved from http://www.arabidopsis.org/. An online platform, https://orthovenn3.bioinfotoolkits.net/, facilitated the execution of comparative genomics analysis. Divergence times for calibration were established using the TimeTree database (http://TimeTree.org). Calibration time points for the study were based on divergence times: *F. hispida* and *M. notabilis* (60 Mya), *F. hispida* and *F. microcarpa* (40 Mya), *F. hispida* and *A. thaliana* (121 Mya).

#### Identification and annotation of variants

SNPs and Indel (length ≤ 500 bp) were compared between two distinct cultivar genomes using MUMmer (v3.23) [89] software. The Nucmer tool within MUMmer was employed initially with parameters ‘-mum -g 1000 -c 90 -l 40’ to generate alignment outcomes. Resultant alignments underwent one-to-one mapping using the delta-filter tool in MUMmer, applying options ‘-r -q’. The Show-snp module in MUMmer, with parameters ‘-ClrTH’, facilitated SNP and Indel identification within the one-to-one alignments. For advanced analysis, the SyRI [90] pipeline was utilized for variant detection. Variant annotation was conducted through the snpEff [91] tool.

Collinearity analysis

To identify homologous gene pairs, we employed BLASTP (v2.2.30+) [83] to calculate pairwise similarities (e-value <1 × 10^−5^), followed by analysis using McscanX [92] with default parameters.

### Transcriptome analysis

#### Preprocessing and alignment

To eliminate low-quality base sequences from original Illumina reads, Trimmomatic v0.388 [67] was employed with default parameters. Bowtie2 (v2.4.5) [93] aligned the processed reads against the SILVA rRNA database of eukaryotes to remove rRNA reads. Unaligned paired reads were aligned to the reference genome using STAR (v2.7) [94] with default parameters. FeatureCounts [95] software calculated transcript counts and TPM values. DESeq2 (v1.28.1) [96] software facilitated differential gene expression analysis. Genes with a false discovery rate (FDR) ≤ 0.05 and |log_2_ fold change| ≥ 2 were defined as DEGs.

#### Identification of pathway enzymes and transcription factors

For the identification of enzyme genes associated with chlorophyll, flavonol, and anthocyanin biosynthetic pathways, we utilized the Plant Metabolic Network [97] and the Ensemble Enzyme Prediction Pipeline (E2P2) package (v3.1) (https://gitlab.com/rhee-lab/E2P2) for querying. The target pathways were established using PlantCyc (https://PlantCyc.org/). Transcription factors within the genome were identified using PlantRegMap [98].

### Co-expression analysis

#### Weighted correlation network analysis

A gene co-expression network was constructed using the R package WGCNA (v1.61) [54] based on the normalized (*z*-score) TPM matrix. RNA-seq data from 21 samples and genes from 12 SRA datasets were included in the network construction. A soft threshold (β) was calculated to achieve a scale-free topology, with a selected scale-free fitting index (R^2^) of 0.9. Gene clustering based on dissimilarity and module definition using the dynamic cut method was performed, with each module containing a minimum of 30 genes sharing similar expression profiles.

Analysis of TO-GCNs

TO-GCN [99] were constructed for purple fruit (ZS5801-specific TO-GCN) and white fruit (ZZB-specific TO-GCN), along with a consensus TO-GCN between the two networks. Potential orthologous genes (34953) were inferred using GeneTribe (v1.2.1) [100]. Orthologous gene pairs were merged as nodes in the consensus TO-GCN. Expressed differentially expressed genes (DEGs) were defined if they displayed significant differential expression between any two of the five fruit ripening time points (S1–S5) and had an average TPM greater than 0.5. Pearson’s correlation coefficient of 0.8 was used for TF-gene pairs. Two bHLH transcription factors (*ZS5801_Monoploid.003909.1* and *ZZB_Monoploid.003862.1*) served as initial nodes for TO-GCNs generated by MFSelector [101] (https://github.com/yushuen/MFSelector_STAR-Protocol). Three TO-GCNs under the ‘C1 + C2+’ mode were combined for an extensive large network and then visualized using Cytoscape [102]. The degrees of the nodes (genes) were calculated as the number of connections or edges the nodes have to other nodes.

Regulatory element analysis

Upstream 2Kb sequences of six core genes (*ZS5801_Monoploid.020731.1*, *ZS5801_Monoploid.012954.1*, *ZS5801_Monoploid.017722.1*, *ZZB_Monoploid.013574.1*, *ZZB_Monoploid.017653.1*, and *ZZB_Monoploid.001452.1*) were extracted, and regulatory element analysis was performed using TBtools [103]. Regulatory subnetworks during the color transition phase (S4 and S5) were plotted for these genes.

#### Variant identification and annotation

Initially, the original reads were pruned using Trimmomatic (v0.38, parameters: LEADING: 30 TRAILING: 30 MINLEN: 75 TOPHRED33) [67]. Subsequently, clean reads underwent quality control and were compared to the ZS5801 haplotype 1 reference genome using BWA-MEM (v0.7.17-r1188-M) [65] with default parameters. Picard (v1.126) (https://broadinstitute.github.io/picard/) was applied to label paired reads aligned to the same reference genome position as replicates and eliminate PCR duplicates. Genetic variation was then detected using the GATK (v4.2.6.1) [104] toolkit: HaplotypeCaller, CombineGVCFs, and GenotypeGVCFs modules of GATK were employed for mutation site acquisition and merging, while the SelectVariants module was used for variant site extraction. BEAGLE (v5.1) [105] software facilitated SNP imputation and phasing. The VariantFlexibility module of GATK was used for variant filtering, including criteria such as mean sequencing depth < 1/3× or > 3×, Quality by Depth (QD) < 2.0, mapping quality (MQ) < 40.0, Fisher Strand (FS) > 60.0, MQRankSum < −12.5, ReadPosRankSum < −8, and retention of only secondary alleles. The final SNP variant set was annotated using SnpEff (v4.3t) [91]. The process for detecting indel variants paralleled that of SNP variants.

#### Phylogeny and population analysis

A comprehensive investigation was conducted into the phylogenetic relationships among mulberry trees, leveraging 4 914 314 high-quality single nucleotide polymorphisms (SNPs). Multiple methods encompassed NJ (neighbor-joining) tree construction, population structure assessment, and genomic SNP-based PCA. The evolutionary tree was generated using Plink (v1.90b6.26) [106] with the ‘—distance-matrix’ parameter for calculating genetic distances. Conversion of results to .meg files was achieved through Perl scripts, and MEGA(v6.0) [107] software was utilized for further analysis (bootstrap = 1000). The resulting tree was visualized using the online tool iTOL [108], with color labels corresponding to sample population information. PCA analysis utilized the EIGENSOFT (v6.1) [109] smartpca program, considering linkage-imbalanced attenuated chromosomal SNPs (plink: —indep-pairwise 50 5 0.4), and significance assessed via the Tracy–Widom test for eigenvalues. PCA plots were generated using ggplot2 in R [110]. Population structure analysis employed ADMIXTURE (v1.3.0) [52] software to scrutinize fruit mulberry’s genetic structure and confounding. ADMIXTURE was executed for k = 2–15 clusters with default parameters. The optimal choice, k = 7, was determined based on minimal CV error, serving as a representative model for the population genetic characteristics of fruit mulberry.

#### Genomic diversity and selection scanning

To assess the attenuation patterns of LD within each population, we calculated the mean r^2^ values between pairs of SNPs within a 500 kb range. These computations were performed using PopLDdecay (v3.27) [111] software, where higher r^2^ values indicate stronger LD. To evaluate genomic differences, we conducted *F*_st_ calculations between two sample groups, employing a sliding window of 25 kb with a step size of 10 kb across the mulberry genome. For this analysis, we utilized VCFtools (v0.1.13) [112], which enabled the quantification of the intensity of genomic differentiation between the two compared groups. The resulting values range from 0 (indicating no differentiation) to 1 (signifying complete or fixed differentiation).

#### GWAS

The GEMMA [53] package, employing mixed linear models, was utilized for the GWAS analysis to enhance statistical power and mitigate false positives. Involving 112 fruit mulberry accessions, GEMMA computed the correlation matrix, and the first three principal component values (feature vectors) derived from high-quality genome-wide SNPs (MAF ≥ 0.05 and deletion rate ≤ 50%) were integrated into the LMM as fixed effects to account for stratification effects. GEMMA generated *P*-values for all SNPs and target traits. The Bonferroni correction established the genome-wide significance threshold of 0.01 divided by the total number of SNPs. Haploblocks were inferred using Plink (v 1.90b6.26) [106] with default parameter (−hap).

#### *VHAG3* selection analysis

For the comparison of *VHAG3* and nucleotide diversity patterns between the purple and non-purple populations, whose population structure is established, we computed *F*_st_, nucleotide diversity (θπ), and Tajima’s *D* using VCFtools (v0.1.13) [112]. These calculations were performed on the SNP dataset using the unit point method, employing a 2 kb window and 1 bp step size.

#### Quantitative real-time PCR

The process of RNA extraction and reverse transcription adhered to previously established protocols [113]. Quantitative real-time PCR (qRT-PCR) was employed to assess the expression of *VHAG3* in mulberry fruit. A 1 μg aliquot of total RNA underwent reverse transcription into cDNA within a 25 μl reaction volume using the PrimeScript RT Kit (TaKaRa). The qRT-qPCR reaction was executed using the SYBR® Premix Ex TaqTM II kit (TaKaRa) and the ABI 7500 real-time quantitative PCR instrument. SYBR qPCR Master Mix was employed for PCR, following the methodology outlined for the StepOne Real-Time PCR System (Applied Biosystems; ABI7500, Foster City, CA, USA). Each sample was prepared with three replicates, with the *ACTIN3* gene as the internal reference gene. The relative expression was computed using the formula 2^-[CT (gene of interest)-CT (internal reference gene)]^ [113]. The primer sequences employed in this study are in Table S18 (see online supplementary material).

### Statistical analysis

The RNA-seq data analysis utilized a negative binomial distribution test to identify differentially expressed genes, with significance determined by a *p-adj* value below 0.05 and a fold change exceeding 2. Metabolite content comparison employed the VIP value, tested using the student’s *t*-test. A VIP value exceeding 1 and a *P*-value below 0.05 indicated statistical significance. In qRT-PCR expression analysis, results were presented as mean ± standard deviation, and intergroup means were compared using the two-sample student’s *t*-test. Significance levels were denoted as follows: ^*^P < 0.05, ^**^P < 0.01, and ^***^P < 0.001. Different lowercase letters were used to indicate significant distinctions between groups.

## Acknowledgements

This research received support from the Earmarked Fund for CARS (CARS-18-ZJ0201), the Chongqing Modern Agricultural Industry Technology System (CQMAITS202311), and the Hainan Province Science and Technology Special Fund (ZDYF2022SHFZ319). We extend our gratitude to Professor Xingtan Zhang from the Institute of Agricultural Genomics, Chinese Academy of Agricultural Sciences, for invaluable comments and suggestions on the manuscript. We also acknowledge the valuable assistance in data analysis provided by Professor Yu Jiang at Northwest A&F University. Furthermore, we appreciate the contributions of Professor Feng Jiao at Northwest A&F University for sharing phenotypic information on germplasm resources and Professor Chunmei Li at Huazhong Agricultural University for providing mulberry fruit metabolism data. We sincerely thank ANNOROAD for its sequencing support and the Hefei Advanced Computing Center for providing computing resources.

## Author contributions

A.Z., Z.X., and W.F. conceptualized and designed the study. M.Z., P.Z., and C.L. prepared the genome sequencing material. A.Z., X.W., Z.S., C.H., and S.W. provided the mulberry materials. Z.X. conducted the genome assembly, while Z.X. and Y.C. performed genome annotation and evaluation. Z.X. and D.L. carried out comparative genomics, population genomics, and selection analyses. Z.X., Y.C., J.L., M.X., and Z.R. conducted transcriptomic analysis. Z.X., D.L., Y.C., J.L., and M.X. were responsible for anthocyanin metabolic pathway reconstruction. Z.X., Y.C., J.L., M.X., and X.W. conducted metabolome and transcriptome co-expression analysis. M.Z. and X.C. cloned the *MaVHAG3* gene, while M.Z. conducted experiments to validate its function. A.Z. and Z.X. wrote the manuscript. All authors reviewed and approved the final version of the manuscript.

## Data availability

The original resequencing reads HiFi data, Hi-C data, RNA-seq data, and Iso-seq data generated during this study have been deposited in the Genome Sequence Archive (GSA) [114] at the National Genomics Data Center (NGDC), Beijing Institute of Genomics (BIG), Chinese Academy of Sciences (CAS) / China National Center for Bioinformation (CNCB) [115] under the accession number CRA011833. These data are publicly accessible at https://ngdc.cncb.ac.cn/gsa. Additionally, the genome assemblies and gene annotations have been deposited in the Genome Warehouse [116] at the NGDC, BIG, CAS / CNCB, with the following accession numbers: GWHDOOQ00000000 (ZS5801_Hap1), GWHDOOP00000000 (ZS5801_Hap2), GWHDOOT00000000 (ZS5801_Monoploid), GWHDOOM00000000 (ZZB_Hap1), GWHDOOO00000000 (ZZB_Hap2), and GWHDOON00000000 (ZZB_Monoploid). These resources are publicly accessible at https://ngdc.cncb.ac.cn/gwh.

## Conflict of interest statement

The authors declare no competing interests.

## Supplementary data

Supplementary data is available at *Horticulture Research* online.

## Supplementary Material

Web_Material_uhae120
