# Supplementary material for: Haplotype-resolved chromosomal-level genome assembly reveals regulatory variations in mulberry fruit anthocyanin content
Source: Hortic Res. 2024 Apr 23;11(6):uhae120. doi: 10.1093/hr/uhae120 (PMC11197311; doi:10.1093/hr/uhae120)
Supplement: Web_Material_uhae120 [file web_material_uhae120.zip › Supplementary_Figure.docx]

**Haplotype-resolved Chromosomal-Level Genome Assembly Reveals Regulatory Variations in Mulberry Fruit Anthocyanin Content**

*Xia et al.*

Supplementary Figures


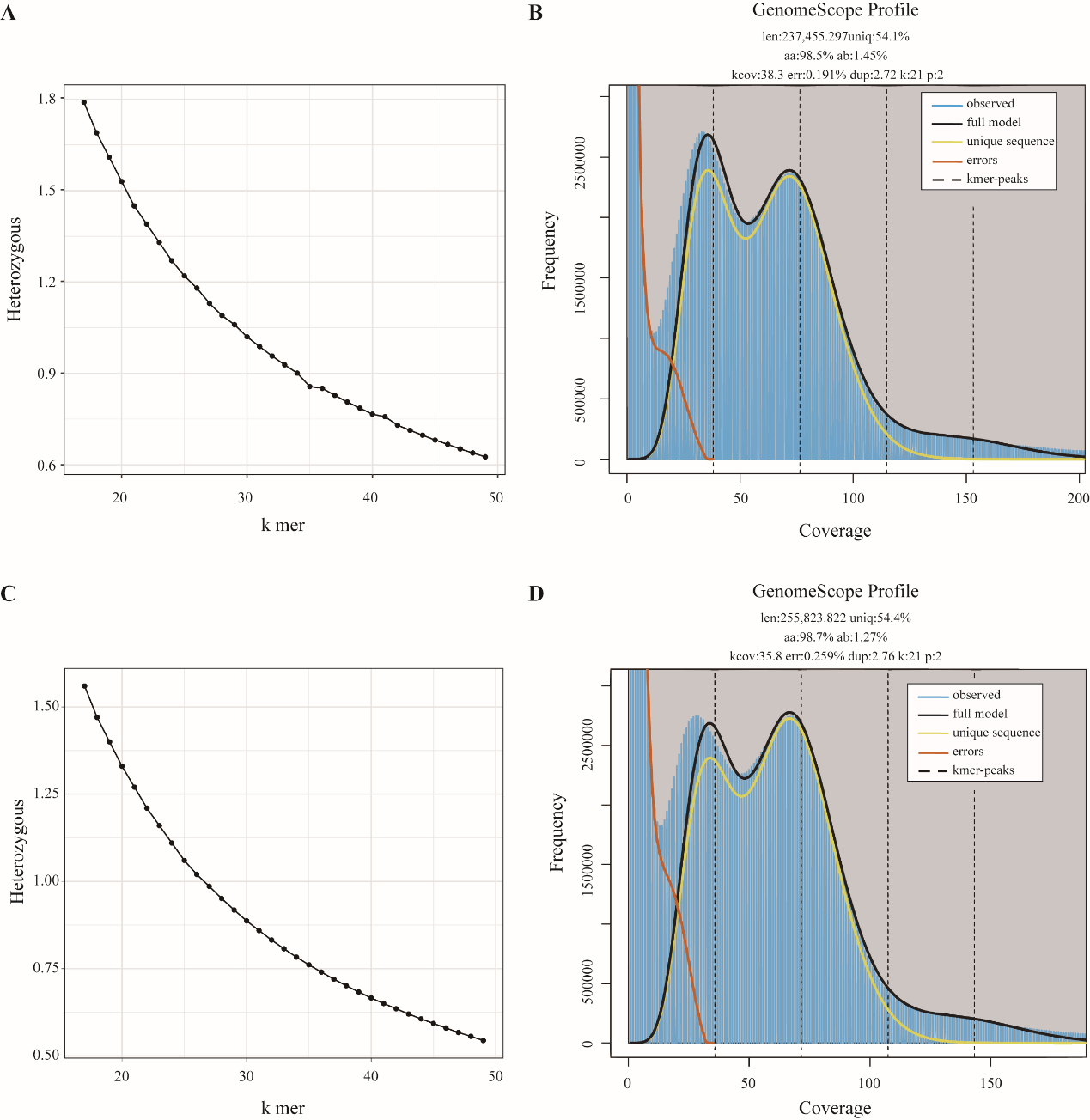


**Figure S1: Genomic heterozygosity evaluation in ZS5801 and ZZB varieties. A** and **B**: Genomic heterozygosity assessment for ZS5801. **C** and **D**: Genomic heterozygosity assessment for ZZB.


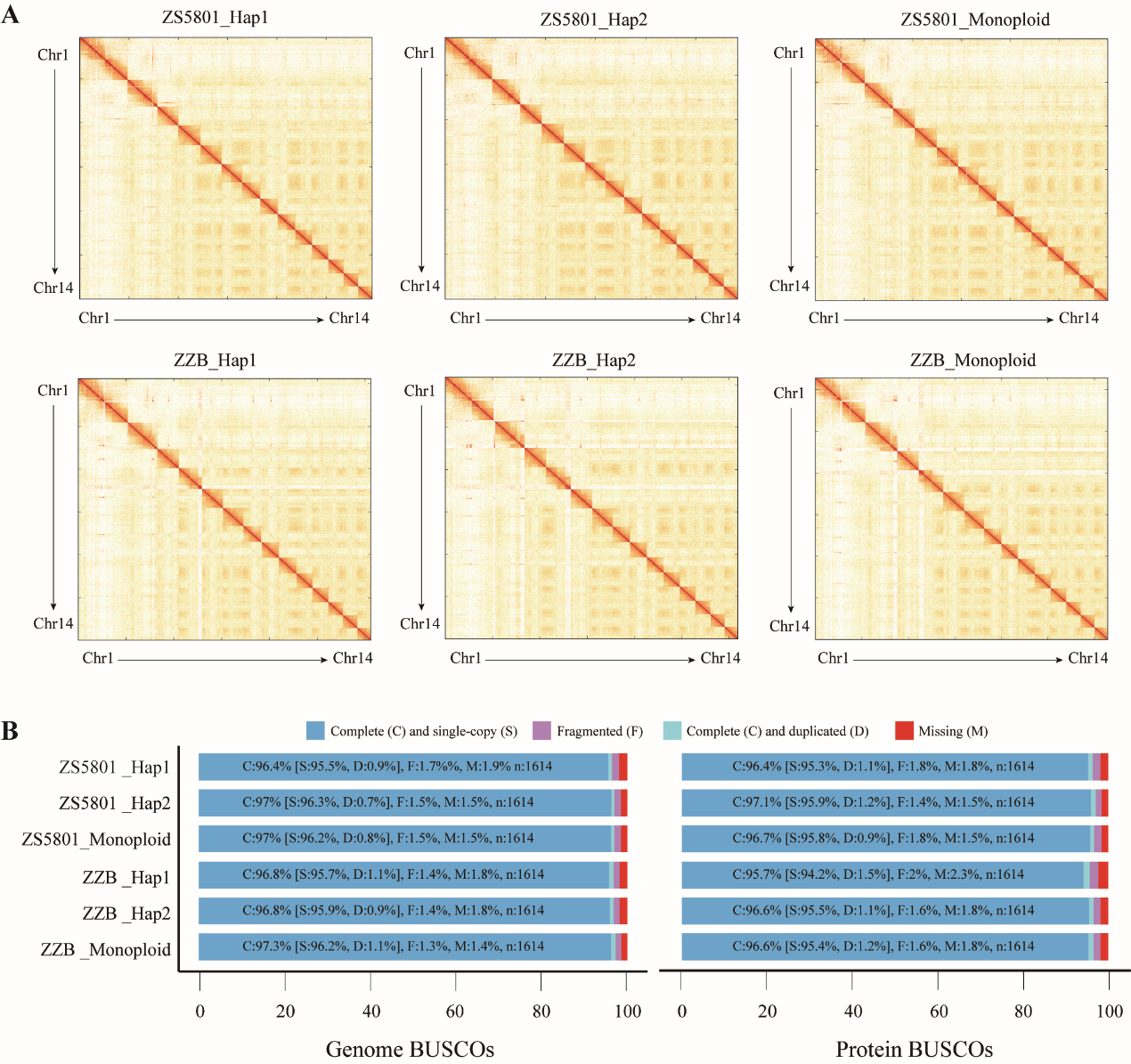


**Figure S2: Comprehensive evaluation of genome assembly in ZS5801 and ZZB. A**: Hi–C chromosome interactions at 100 kb resolution within ZS5801 and ZZB. Intense interactions are denoted in deep red, while feeble interactions are delineated in yellow. **B**: Evaluation of ZS5801 and ZZB genome assembly and annotation quality through BUSCO analysis.


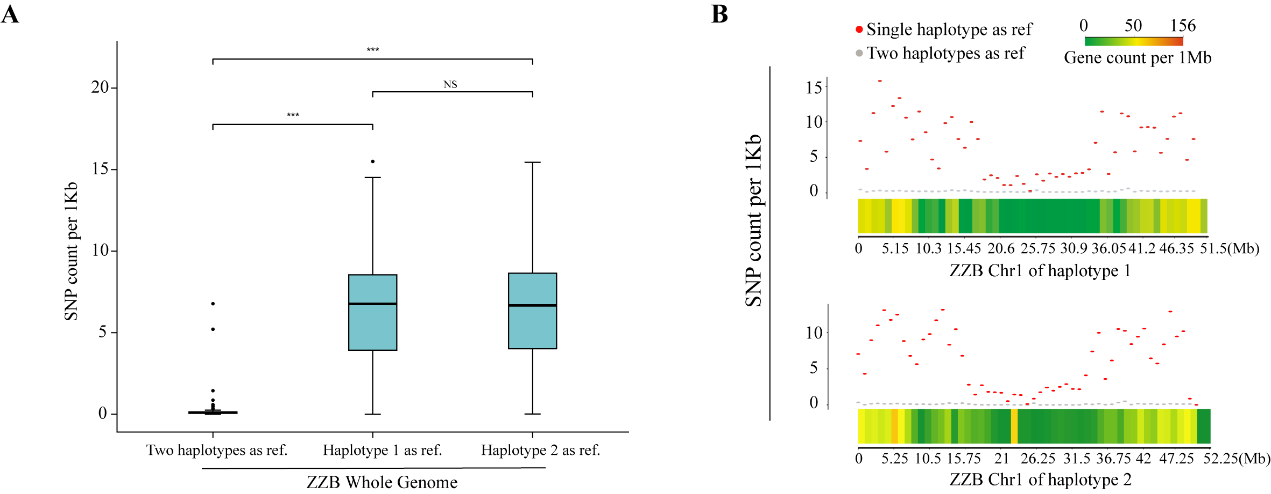


**Figure S3: Analysis of haplotype dominance in ZZB.** **A**: Assessment of SNP Density Utilizing Varied Haplotype References. This analysis comprises three sequential boxplots. The initial boxplot illustrates SNP density within a 1 Kb window, utilizing the amalgamation of two haplotypes as a reference. Subsequently, the second and third boxplots individually showcase SNP densities for haplotypes 1 and 2, employing a sole haplotype as a reference. Notably, aligning reads to distinct allelic segments and identifying heterozygous sites as SNPs amplifies the SNP density grounded on a singular haplotype reference. **B**: Chromosome 1-specific distribution of SNP density using diverse ZZB reference genomes. This segment presents a visualization of the SNP density distribution for the two haplotypes on chromosome 1 in ZZB by applying diverse reference genomes.


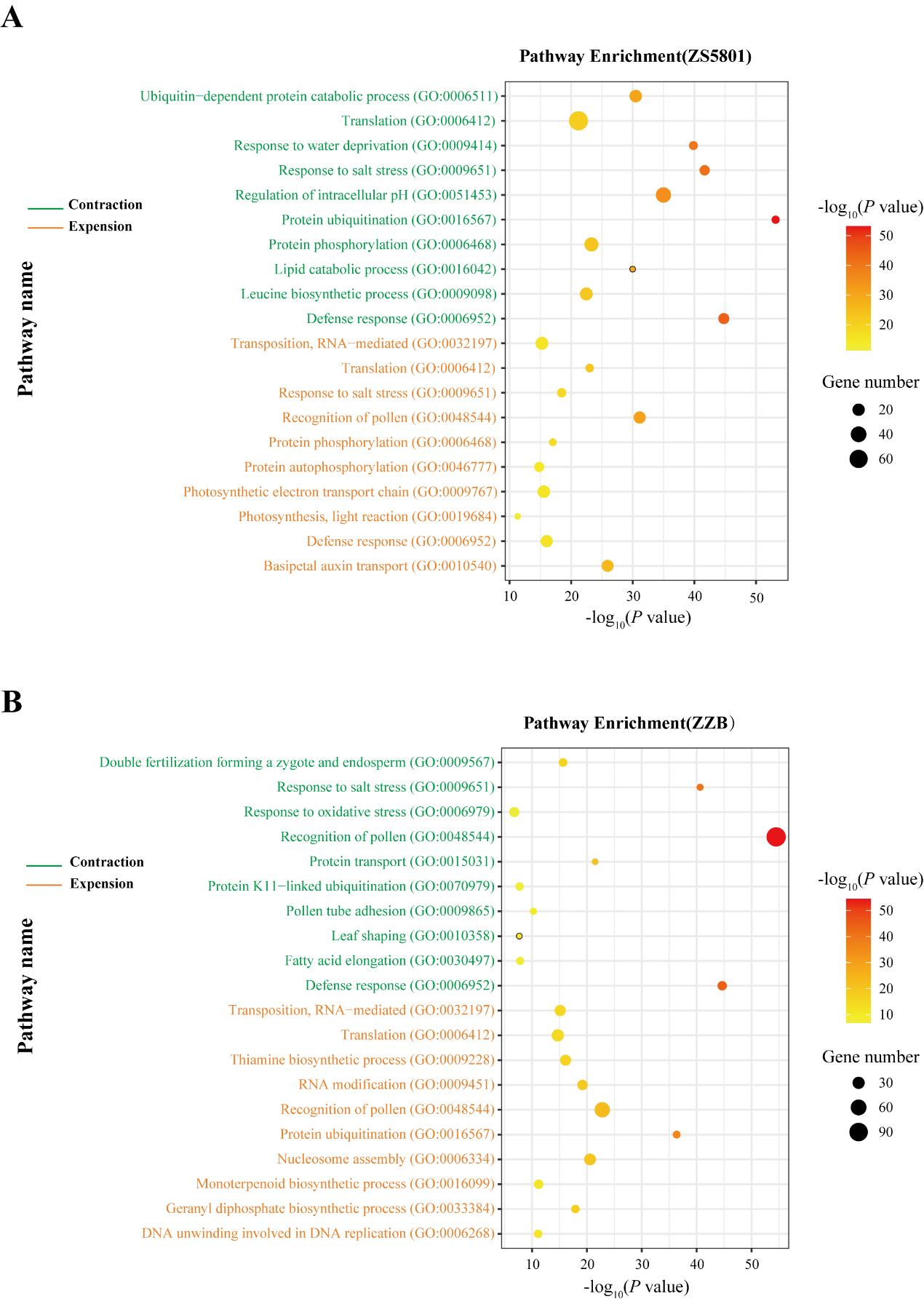


**Figure S4: GO annotation of gene families expands and contracts ZS5801 and ZZB. A**: GO annotation of gene family expansion and contraction in ZS5801. This section presents the GO annotation detailing gene family expansion and contraction in ZS5801. The analysis identified 70 expanded and 183 contracted gene families. The expanded gene families are prominently associated with biological processes, including protein ubiquitination, ATP-active enzymes, and defense responses. **B**: GO annotation of gene family expansion and contraction in ZZB. GO annotation elucidates gene family expansion and contraction in ZZB in this segment. Specifically, 76 significantly expanded gene families and 142 contracted gene families were highlighted. Remarkably, the expanded gene families primarily engage in processes related to pollen recognition and salt stress response.


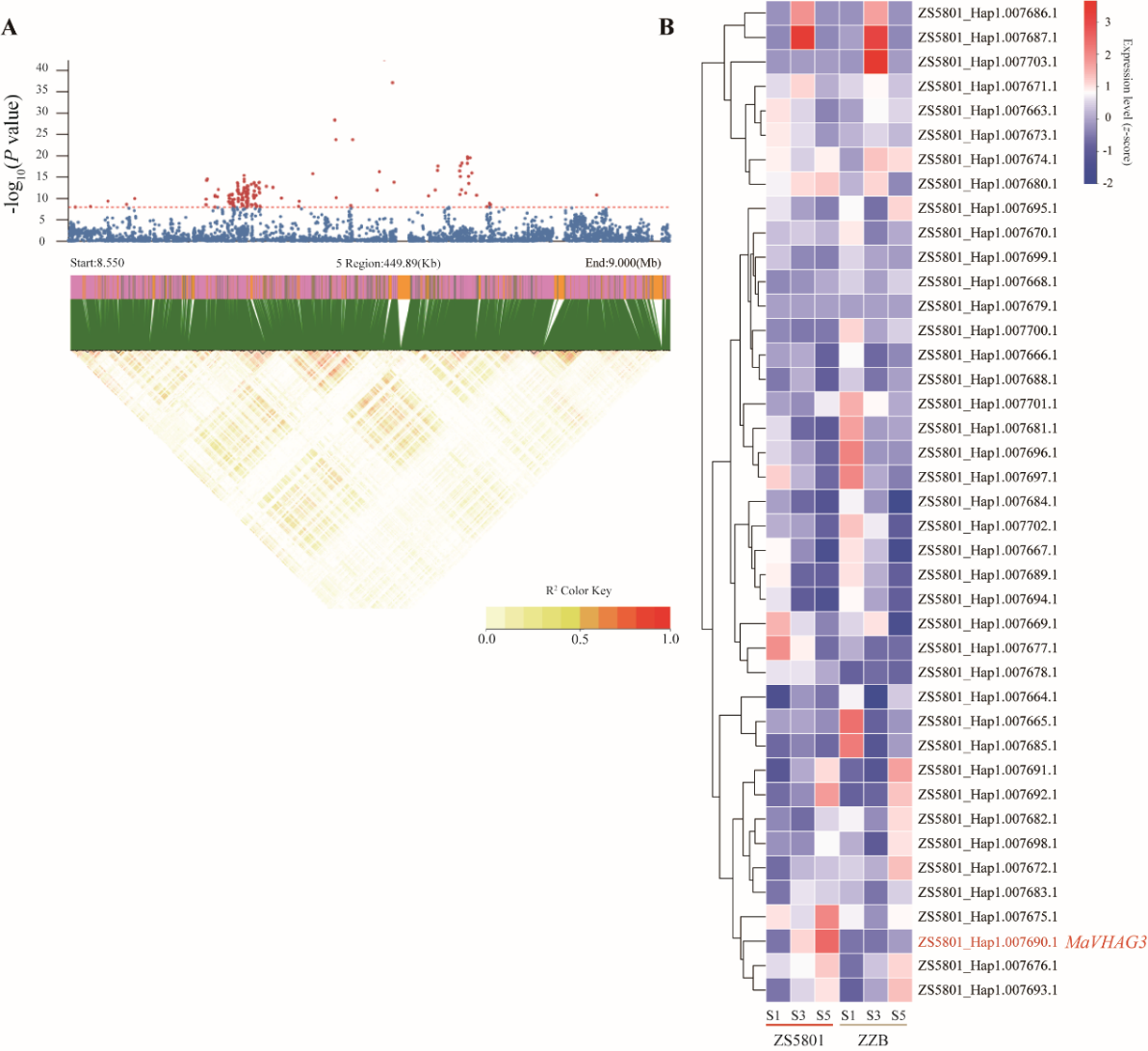


**Figure S5: GWAS candidate region.** **A**: Detailed plot showing the genomic region (8.55-9.00 Mb) surrounding the lead SNP (red dot) on chromosome 5. The color of each dot corresponds to the r^2^ value, as the color scale indicates, representing the pairwise linkage disequilibrium values among all polymorphic sites. **B**: Gene expression profiles of the region neighboring the marker-trait association (MTA) at each stage of fruit development. *MaVHAG3* exhibited significantly higher expression levels in ZS5801, particularly during fruit ripening.


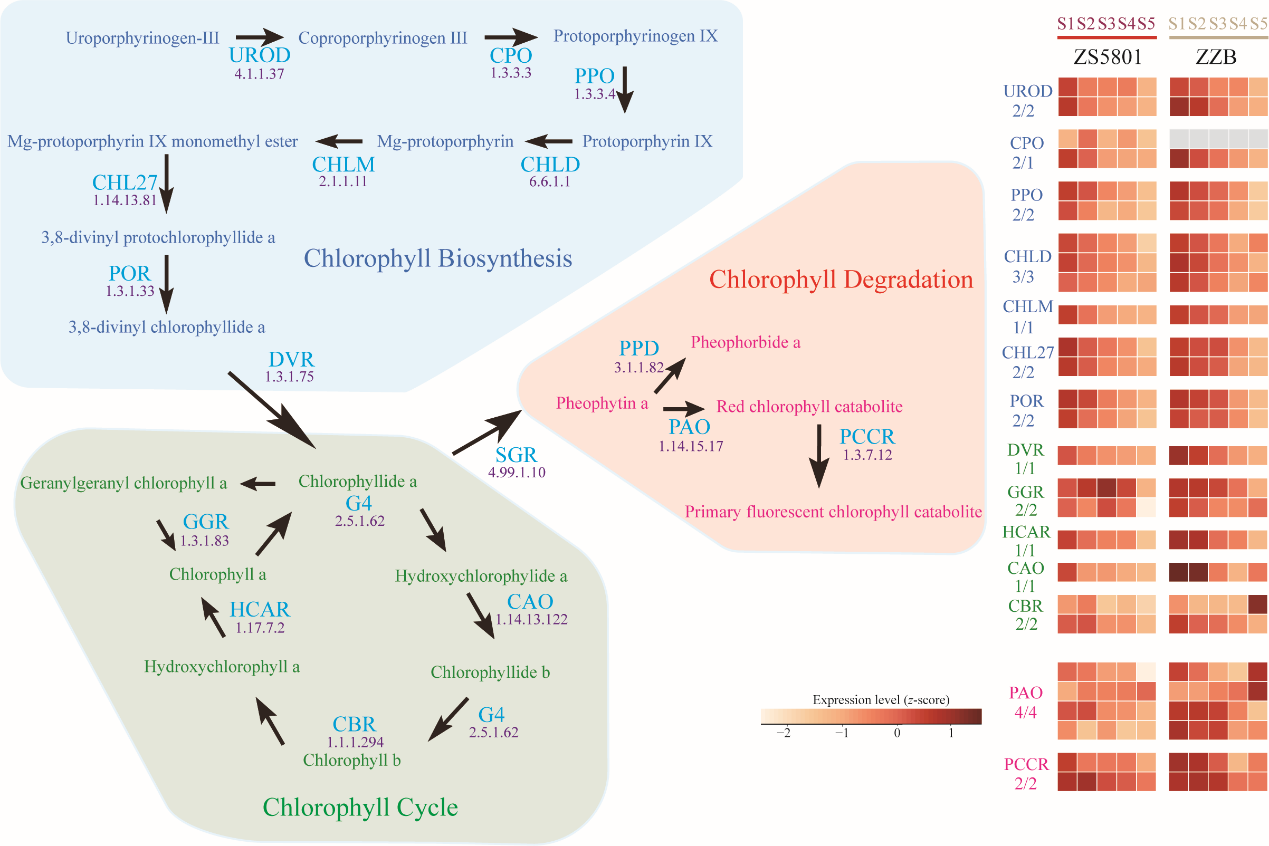


**Figure S6: Expression analysis of the chlorophyll metabolism pathway**. Heat map depicting gene expression values obtained from RNA-seq data at various fruit development stages (S1–S5) for the ZS5801 and ZZB cultivars. The chlorophyll enzyme genes are classified into three distinct groups: “chlorophyll biosynthesis,” “chlorophyll cycle,” and “chlorophyll degradation.” The genes encoding the following enzymes are represented: UROD (uroporphyrinogen decarboxylase), CPO (coproporphyrinogen oxidase), PPO (protoporphyrin oxidase), CHLD (magnesium chelase), CHLM (magnesium protoporphyrin IX methyltransferase), CHL27 (magnesium-porphyrin IX 13-monomethyl ester cyclase), POR (photo-independent 3,8-divinyl-protochlorophyll reductase), DVR (3,8-Divinylchlorophyll 8-vinyl reductase), GGR (geranyl chlorophyll A reductase), HCAR (7-hydroxychlorophyll A reductase), CAO (chlorophyll-a: oxygen 7-oxidoreductase), CBR (chlorophyll B reductase), PAO (pheophorbide an oxygenase), and PCCR (red chlorophyll catabolite reductase).


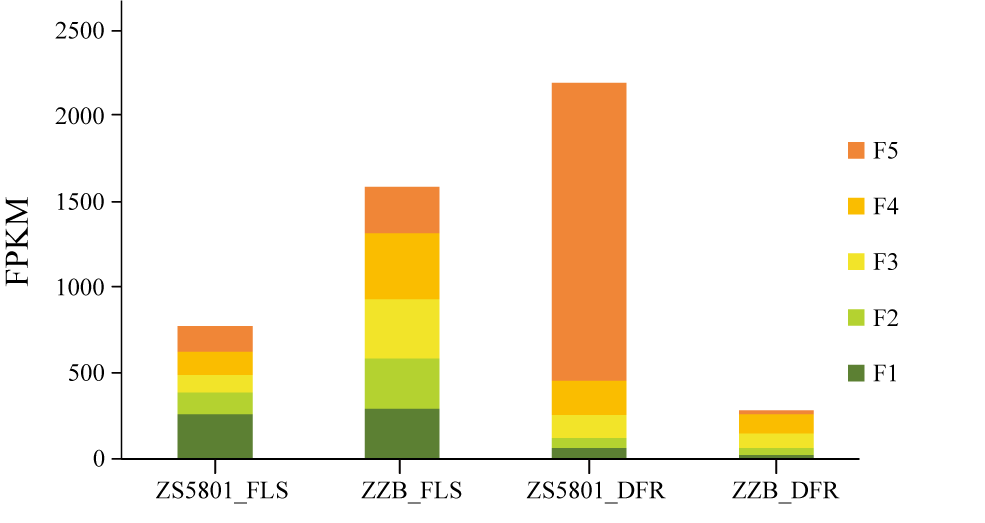


**Figure S7: Total expression of all FLS and DFR genes.** Stacked histograms illustrating the total expression of FLS (8:12) and DFR (7:5) genes in ZS5801 and ZZB at various stages of fruit development. The data revealed a significant decrease in total expression for the contracted DFR gene, whereas the total expression of the expanded FLS gene exhibited a substantial increase.


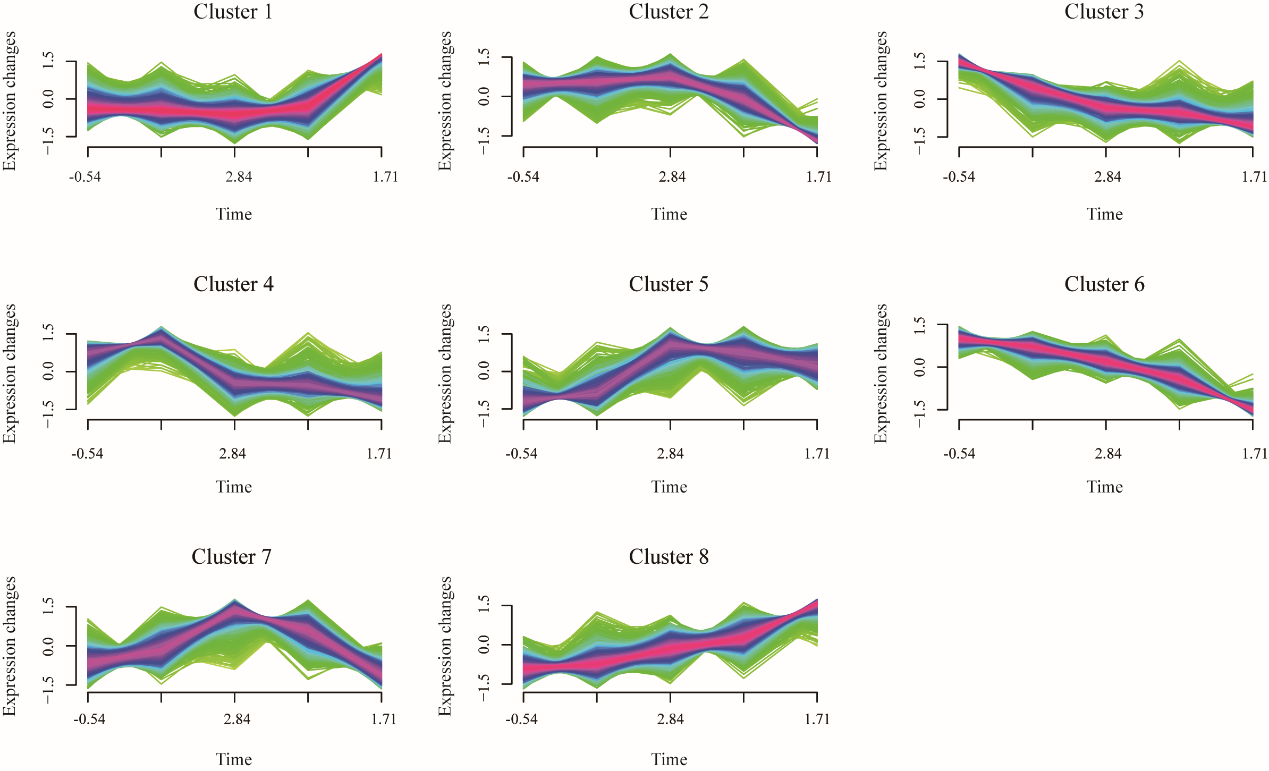


**Figure S8: DEG gene trend clustering.** Trend clustering of gene expression revealed eight distinct clusters representing genes with diverse expression patterns. These clusters are further segregated into two trends based on variety: “Similar” and “Opposite.”


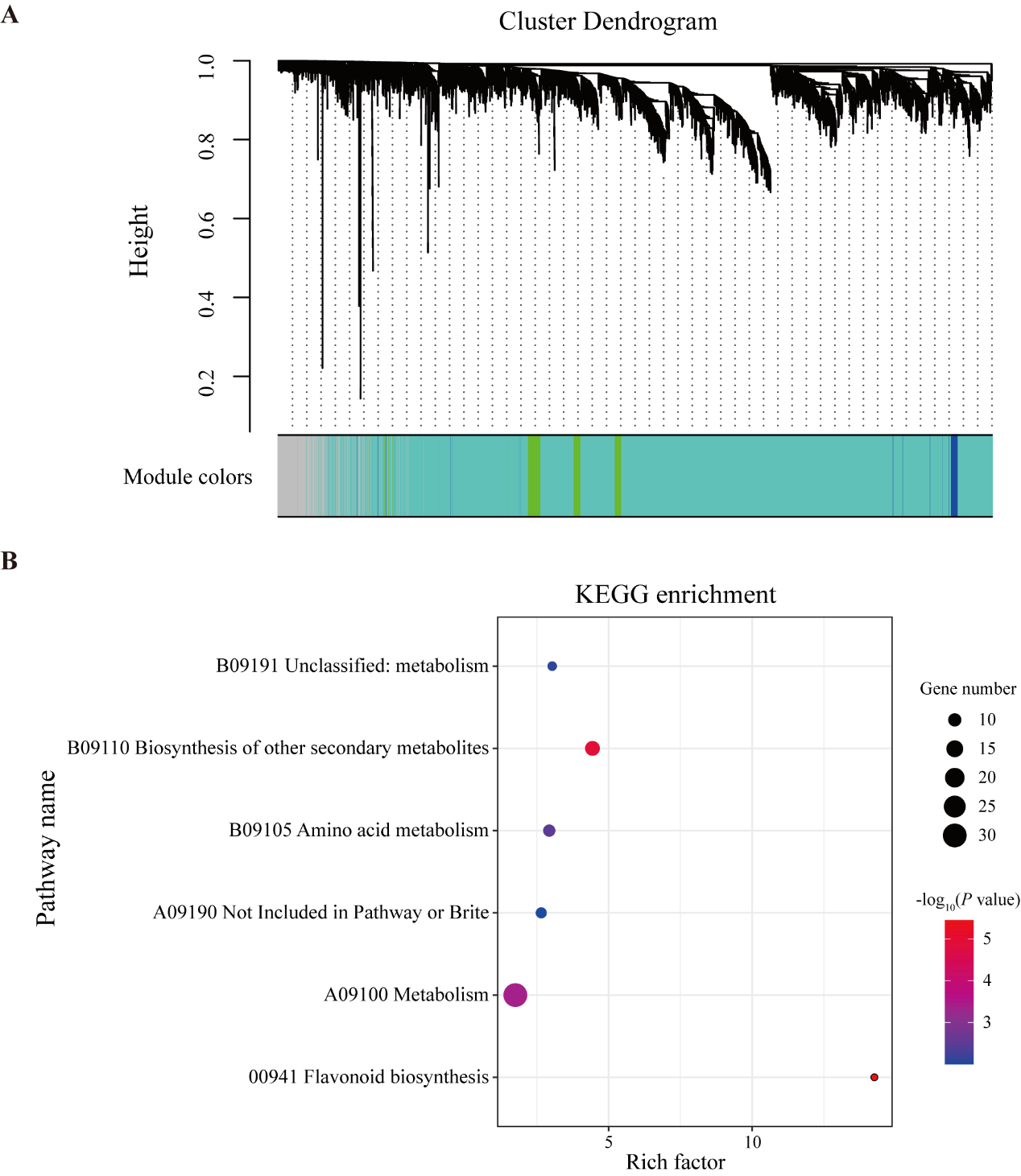


**Figure S9: WGCNA analysis.** **A**: Dendrogram illustrating the hierarchical clustering of gene modules. In total, 23 modules comprising 46,541 genes were constructed. **B**: Functional enrichment analysis of genes within the salmon module identified through WGCNA. The salmon module, consisting of 110 genes, includes several well-known key genes involved in the anthocyanin biosynthetic pathway. Notably, these genes exhibited significant functional enrichment in flavonoid synthesis, suggesting their potential association with color accumulation.


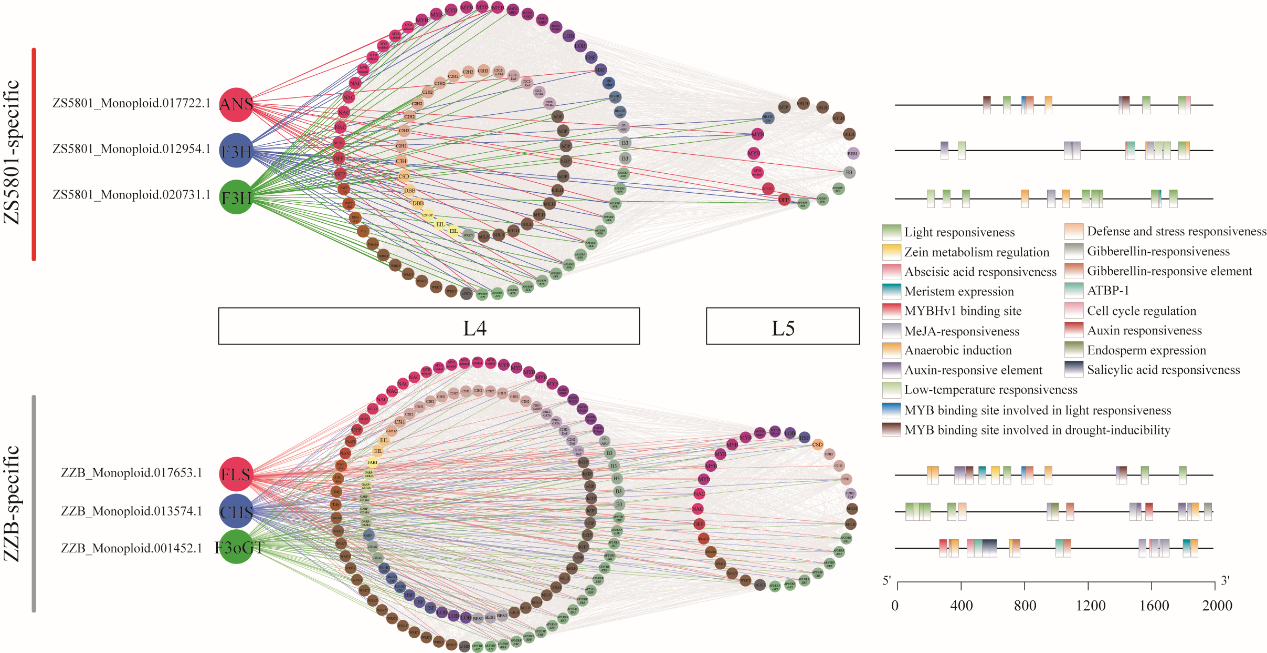


**Figure S10: Promoters and subnetworks of a high degree in L4 and L5.** **A**: Promoter regulatory elements identified within the upstream 2 Kb sequences of six core genes during the color transition (S4 and S5). **B**: Regulatory subnetworks associated with the identified core genes highlight their intricate interactions and potential regulatory pathways during the color transition process.


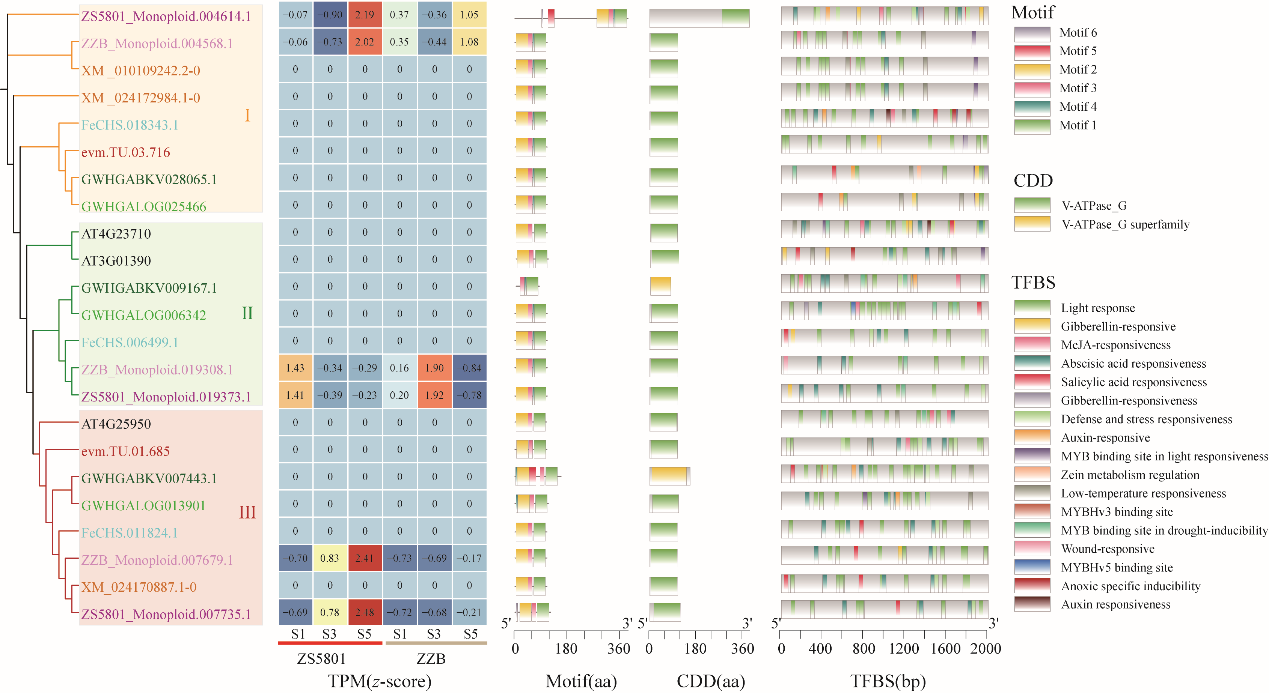


**Figure S11: The *VHAG* gene family evolution.** This figure showcases five annotations arranged from left to right, comprehensively depicting the *VHAG* gene family across eight distinct species through a detailed phylogenetic tree. It also includes meticulous identification of gene expression profiles, thorough validation of gene structures using Motif and CDD analysis, and insightful predictions of transcription factor binding sites within the 2 kb upstream region of the genes.
